# Bioactive polythioctic acid-engineered ropivacaine alleviates bone cancer pain through prolonged analgesia and multi-functional neuroprotection

**DOI:** 10.1016/j.mtbio.2026.103693

**Published:** 2026-09-18

**Authors:** Ziping Wu, Yucen Zhang, Yan Guo, Yueying Wang, Zhen Wang, Zhuo Yang, Dongmei Guo, Chao Xu, Boyuan Gu, Haoyu Zheng, Yali Yan, Ming Zhang, Dan Shao, Li Chen, Aitao Wang

**Affiliations:** a College of Basic Medical Sciences, The Medical Basic Research Innovation Center of Airway Disease in North China, Key Laboratory of Pathobiology, Ministry of Education, Jilin University, Changchun, 130021, China; b Hohhot First Hospital, Hohhot, Inner Mongolia, 010030, China; c Hohhot Stomatological Hospital, Inner Mongolia Autonomous Region Stomatological Hospital, Hohhot, Inner Mongolia, 010110, China; d Department of Anesthesiology, The Fifth Clinical Medical College, Inner Mongolia Medical University, Hohhot, Inner Mongolia, 010030, China; e State Key Laboratory of Oral Diseases and National Clinical Research Center for Oral Diseases and Department of Oral and Maxillofacial Surgery, West China Hospital of Stomatology, Sichuan University, Chengdu, Sichuan, 610041, China; f Department of Anesthesiology, Inner Mongolia People's Hospital, Hohhot, Inner Mongolia, 010017, China

**Keywords:** Bone cancer pain, Ropivacaine, Polythioctic acid, Neuroprotection, Sustained analgesia

## Abstract

Bone cancer pain (BCP), driven by tumor-induced bone destruction and central sensitization, represents a major clinical challenge. Although ropivacaine (Rop) is a cornerstone for BCP management, it fails to provide prolonged analgesia and neuroprotective benefits. Through RNA transcriptomics and phenotypic analysis in a murine model of metastatic BCP, we show that while Rop rapidly alleviates mechanical and thermal hyperalgesia, it paradoxically exacerbates spinal oxidative stress and neuroinflammation. To address this paradox, we engineered a multifunctional, redox-responsive polymer platform designed to prolong analgesia and mitigate Rop-associated neuronal injury concurrently. Leveraging polythioctic acid (PTA) with a responsive disulfide framework, the encapsulated Rop achieves a sustained release profile to facilitate prolonged analgesia under reductive tumor microenvironment. Meanwhile, the liberated thioctic acid (TA) acts as an antioxidant to ameliorate both neurotoxicity and neuroinflammation. Consequently, the PTA@Rop nanoparticle achieves a 1.6-fold extension of the analgesia window compared to free Rop *in vivo*, leading to preservation of bone microarchitecture and suppression of pro-inflammatory cytokine cascades. Mechanistically, transcriptomic profiling further reveals that PTA@Rop platform reshapes the spinal gene expression landscape and reduced nerve growth factor (NGF) homeostasis to suppress central sensitization. Our nanobiotechnology engineering decouples prolonged analgesia of Rop from unwanted neurotoxicity, providing proof-of-concept evidence for a mechanism-driven nanoplatform for BCP management.

## Graphical abstract

**Figure:**
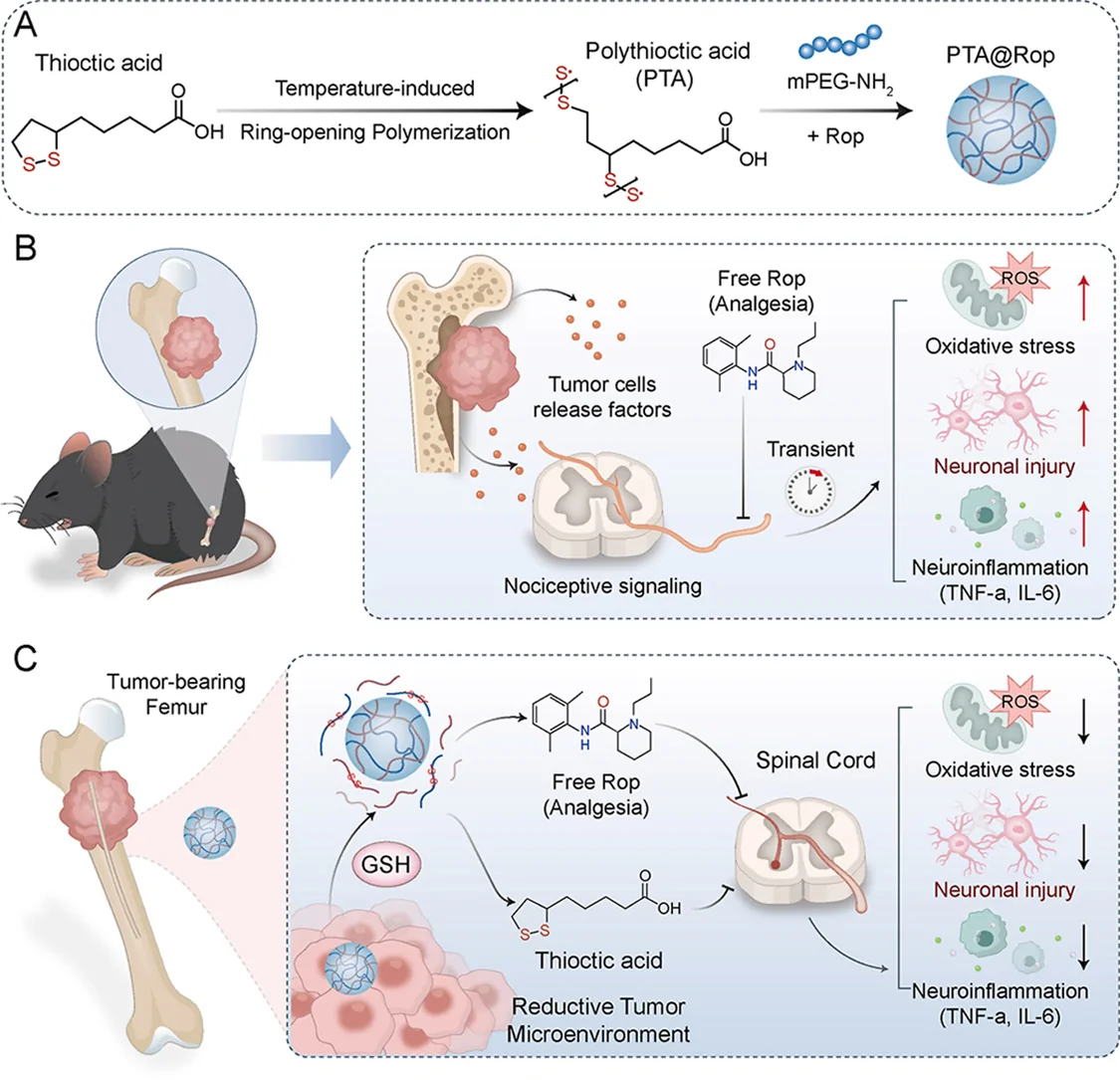

## 1 Introduction

Bone cancer pain (BCP) is a severe complication commonly associated with bone metastasis from primary tumors, such as lung and breast cancers [[1], [2], [3]]. BCP is characterized by progressive bone destruction, mechanical allodynia, and central sensitization [4]. Current treatments, including opioids, surgery, and local anesthetics, mainly provide symptomatic pain relief [5,6]. However, these treatments cannot fully control the pathological processes of BCP, especially persistent neuroinflammation and oxidative stress [[7], [8], [9], [10]]. Bone-targeted medicines, such as bisphosphonates and denosumab, remain important components of multimodal BCP treatment. Therefore, a localized nanoplatform should be considered as a potential adjunct to, rather than a replacement for, performing the combination of oncological and analgesic treatments. The development of new therapeutic strategies that provide prolonged analgesia while regulating the neuroinflammation is a huge challenge.

Ropivacaine (Rop) is a long-acting amide-type local anesthetic widely used for perioperative regional anesthesia and pain management [[11], [12], [13], [14], [15]]. However, the effective duration of a single local administration remains limited, and repeated injections may be required to maintain analgesia [16,17]. Under the experimental exposure conditions in animal studies, free Rop administration was always accompanied by neuronal injury, oxidative stress, and increased spinal inflammatory signaling [18,19]. These findings do not indicate that Rop causes neurotoxicity during clinical use [20]. Instead, they suggest that prolonged local exposure without neuroprotective support may produce additional neuronal stress and promote neuroinflammation, thereby contributing to central sensitization under the tested BCP conditions [21,22].

To address this problem, a multifunctional biomaterial platform that combines prolonged analgesia with neuroprotection is required [[23], [24], [25], [26], [27]]. We hypothesized that simultaneous regulation of local anesthetic release and spinal pathological activation could provide an improved strategy for BCP management [28,29]. Based on the unique 1,2-dithiolane ring structure of thioctic acid (TA), we synthesized polythioctic acid (PTA) through temperature-induced ring-opening polymerization [30]. PTA serves as both a drug carrier and a bioactive therapeutic component. Its disulfide-rich framework can undergo reductive cleavage in the pathological microenvironment, thereby promoting Rop release. Meanwhile, PTA depolymerization releases bioactive TA, which can reduce oxidative stress and suppress neuroinflammation. Compared with conventional inert carriers that mainly act as drug reservoirs, PTA serves not only as a reduction-responsive carrier matrix, but also as a bioactive precursor. The cleavage of the disulfide-rich backbone of PTA promotes the release of Rop and simultaneously regenerate TA with antioxidant activity. Moreover, the introduction of the mPEG segment is beneficial for the self-assembly of nanoparticles in aqueous solution and the formation of a hydrated outer layer, thus providing steric stabilization. Therefore, we hypothesize that the combination of drug delivery capacity and therapeutic activity offers reasonable benefits of the selection of PTA for mechanism-driven management of BCP.

Herein, we developed a multifunctional PTA-based nanoparticle platform, termed PTA@Rop, for the treatment of BCP [[31], [32], [33]]. Through its reduction-responsive disulfide framework, PTA@Rop enabled sustained local release of Rop and prolonged the analgesic duration in a murine metastatic BCP model (Scheme 1). At the same time, the released TA reduced Rop-associated oxidative stress and ameliorated spinal neuroinflammation [[34], [35], [36]]. Transcriptomic, histological, and behavioral analyses further showed that PTA@Rop regulated spinal inflammatory and neuronal signaling pathways and restored nerve growth factor (NGF) expression toward normal levels, thereby reducing central sensitization [37,38]. Collectively, this study presents a bioactive engineering strategy that combines prolonged local analgesia with multi-functional neuroprotection for BCP management.

**Scheme 1:**
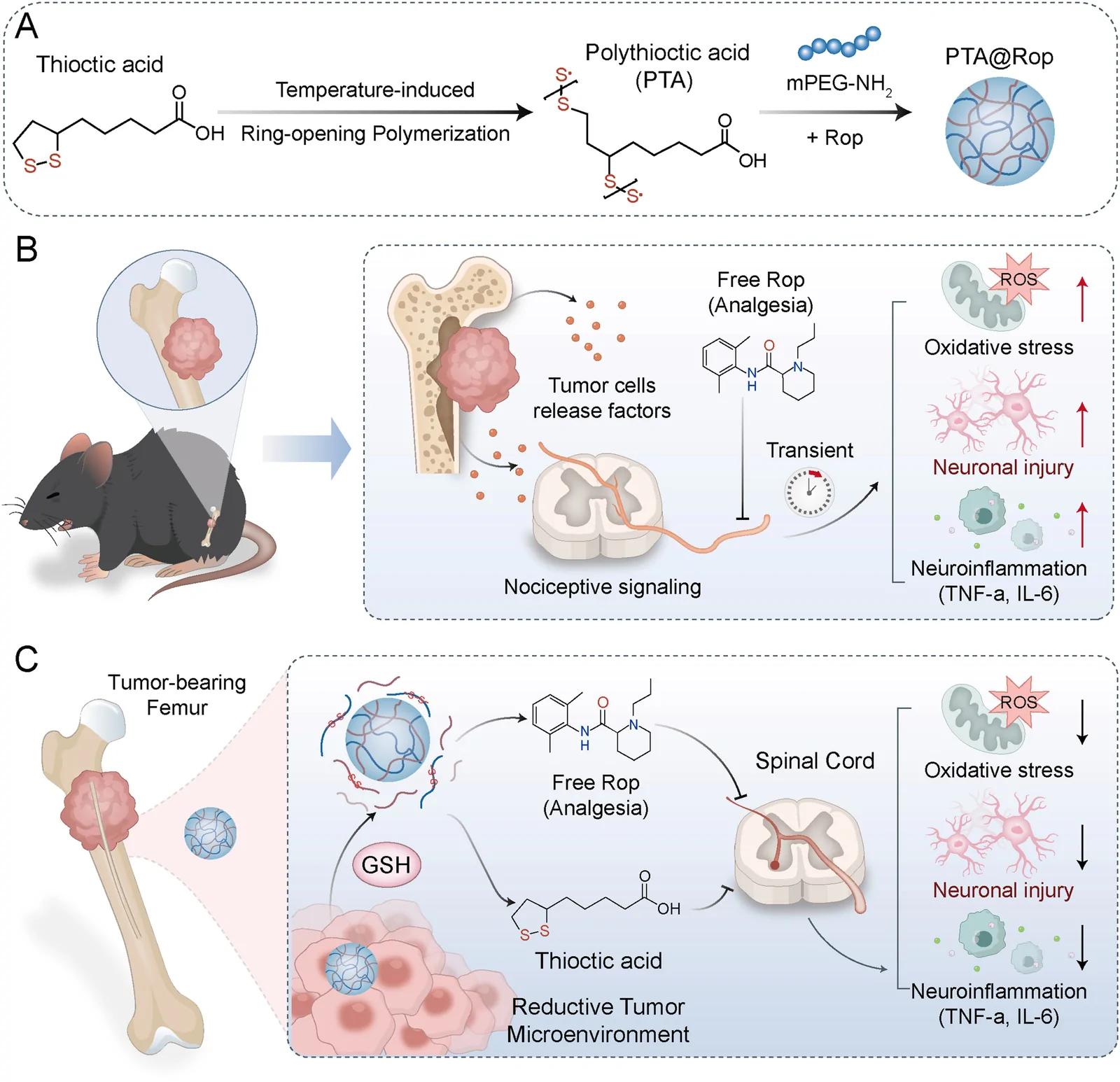
Schematic illustration of the multifunctional PTA@Rop nanoplatform for BCP management. (A) Synthesis of PTA and preparation of PTA@Rop nanoparticles. (B) Pathophysiology of BCP and the accompanying paradox of Rop-induced neurotoxicity, involving tumor-derived nociception, oxidative stress, neuronal injury, and neuroinflammation. (C) Mechanism of action within the reductive BCP microenvironment, wherein elevated glutathione (GSH) triggers the responsive cleavage of disulfide bonds within PTA@Rop, achieving synchronized Rop release for extended analgesia while liberating bioactive TA. The regenerated TA acts as an intrinsic neuroprotective shield that mitigates oxidative damage, suppresses inflammatory cascades, and reshapes the spinal transcriptomic landscape.

## 2 Experimental section

### 2.1 Materials

Thioctic acid (TA) and ropivacaine hydrochloride hydrate (Rop) were purchased from Adamas (Guangzhou, China) and Aladdin (Shanghai, China), respectively. Neurobasal medium, B27 supplement, fetal bovine serum (FBS), Dulbecco's modified Eagle's medium (DMEM), and penicillin–streptomycin were obtained from Gibco (Thermo Fisher Scientific, USA). ELISA kits for TNF-α and IL-6 were purchased from BioLegend (San Diego, CA, USA). Commercial kits for detecting reactive oxygen species (ROS), malondialdehyde (MDA), superoxide dismutase (SOD), and glutathione (GSH) were obtained from Nanjing Jiancheng Bioengineering Institute (Nanjing, China). All other reagents were of analytical grade and used without further purification.

### 2.2 Synthesis of PTA and mPEG-PTA

Polythioctic acid (PTA) was synthesized by temperature-induced ring-opening polymerization. Briefly, TA (2.06 g, 10 mmol) was heated at 80°C under a nitrogen atmosphere for 2 h. The obtained crude product was dissolved in tetrahydrofuran and recrystallized using cold diethyl ether to obtain purified PTA. For the preparation of mPEG-PTA, PTA was conjugated with mPEG-NH_2_ (2.0 g, 1 mmol) using EDC·HCl (383 mg, 2 mmol) and DMAP (61.08 mg, 0.5 mmol) in a THF/DMSO mixture (1:1, v/v). The reaction mixture was stirred at room temperature for 48 h, dialyzed against deionized water for 3 days using a dialysis membrane with a molecular-weight cutoff of 3500 Da, and then lyophilized to obtain mPEG-PTA.

### 2.3 Preparation of PTA@Rop reduction-responsive nanoparticles

PTA@Rop nanoparticles were prepared by a nanoprecipitation and self-assembly method. Briefly, mPEG-PTA and Rop were co-dissolved in N,N-dimethylformamide (DMF). PBS (pH 7.4) was then slowly added dropwise under magnetic stirring in the dark to induce nanoparticle formation. After stirring for 2 h, the mixture was dialyzed against deionized water to remove residual organic solvent and unencapsulated Rop. The final product was obtained by lyophilization. Rop-loaded PLGA nanoparticles (PLGA@Rop) were prepared as a non-redox-responsive carrier control.

### 2.4 Physicochemical characterization of nanoparticles

The hydrodynamic diameter, PDI and zeta potential of the nanoparticles were measured using a Malvern Zetasizer Nano ZS. The morphology of PTA and PTA@Rop was observed by transmission electron microscopy (TEM). UV–visible absorption spectra were recorded using a UV-3600 Plus spectrophotometer, and Fourier-transform infrared spectra were obtained using a Bruker TENSOR II spectrometer. The number-average molecular weight (Mn) and dispersity (Đ) of PTA were measured by gel permeation chromatography (GPC). The size stability of PTA@Rop in PBS and 10% FBS was monitored at 37°C for 7 days. Drug-loading content (DL) and encapsulation efficiency (EE) were calculated using the following equations:

EE(%)=(massofRopencapsulatedinPTA@Rop/massofRopadded)×100%

DL(%)=(massofRopencapsulatedinPTA@Rop/totalmassofdrug−loadedPTA@Ropnanoparticles)×100%

### 2.5 *In vitro* drug releasing behavior of PTA@Rop

The *in vitro* release profiles of ropivacaine (Rop) and thioctic acid (TA) from PTA@Rop were evaluated in phosphate-buffered saline (PBS, pH 7.4) and a reducing medium containing 10 mM glutathione (GSH). Briefly, PTA@Rop was immersed in the release medium and incubated at 37°C in the dark. At designated time points, 1 mL aliquots of the supernatant were withdrawn and centrifuged, and the released amounts of TA and Rop were determined via UV-Vis spectrophotometry. To minimize experimental error, the supernatant was returned to the release system after each measurement. The drug loading capacity and encapsulation efficiency were calculated based on the volume and concentration measured at each time point. For the non-redox-responsive control, PTA@Rop and PLGA@Rop with matched Rop contents were incubated in PBS with or without 10 mM GSH at 37°C. Their hydrodynamic diameter, PDI, and Rop-release profiles were compared under the same conditions.

### 2.6 Cell culture

Lewis lung carcinoma cells, 4T1 breast cancer cells, Neuro-2a (N_2_A) cells, L929 mouse fibroblasts, and C2C12 myoblasts were obtained from the indicated commercial cell banks. Cells were cultured in DMEM containing 10% FBS and 1% penicillin–streptomycin at 37°C in a humidified atmosphere containing 5% CO_2_.

### 2.7 Isolation and culture of primary cells from rat dorsal root ganglia (DRG)

Lumbar dorsal root ganglia (DRG) were harvested from euthanized three-week-old Sprague-Dawley (SD) rats under sterile conditions. Following removal of connective tissue, the DRG were digested with collagenase and trypsin, dissociated into a single-cell suspension, and seeded onto poly-L-lysine-coated plates. The cultured neurons were subsequently maintained in Neurobasal medium supplemented with B27 for further experiments.

### 2.8 Cell viability assay

Cell viability was evaluated by the MTT assay. Briefly, cells were seeded in 96-well plates at a density of 1 × 10^4^ cells/well and allowed to attach for 12 h. Following 12 h of exposure to various drug concentrations, the medium was replaced with fresh DMEM, and the cells were incubated for an additional 24 h. MTT solution (100 μL, 5 mg/mL) was added to each well. After 4 h of incubation, the medium was discarded, and 100 μL of DMSO was added to dissolve the formazan crystals. The absorbance was finally measured at 570 nm using a microplate reader.

### 2.9 Flow-cytometric apoptosis assay

Cell apoptosis was detected using an Annexin V-FITC/PI apoptosis kit. After treatment, DRG neurons were collected, washed with PBS and resuspended. The cells were stained with Annexin V-FITC and PI for 5 min at room temperature in the dark and then analyzed using a flow cytometer (BD Biosciences, USA). Approximately 5 × 10^3^ cell events were collected for each sample. The same acquisition settings and gating strategy were used for all groups.

### 2.10 Establishment of plantar wound incision model

Mice were anesthetized with sevoflurane, after which a 5 mm longitudinal incision was made on the plantar surface of the left hind paw with incision of the underlying plantaris muscle. The wound was closed using 5-0 nylon sutures, followed immediately by peri-incisional injection of 0.1 mL of PTA@Rop. Baseline mechanical and thermal withdrawal thresholds were recorded, with additional measurements taken at 0.5, 2, 4, 6, 8, 24 and 48 h post-injection.

### 2.11 Establishment of bone cancer pain (BCP) model

Lewis lung carcinoma cells were digested and resuspended in PBS at 2 × 10^7^ cells/mL. For tumor inoculation, mice anesthetized with 2% isoflurane received a incision near the knee joint to expose the patellar ligament. A 25-gauge needle was inserted into the femoral medullary cavity via the intercondylar notch, then replaced with a 10 μL microinjection syringe containing 2 × 10^7^ cells. The cells were slowly injected into the medullary cavity over 2 min. The injection site was sealed with bone wax to prevent extravasation, and the skin incision was closed. Mice with failed inoculation or postoperative motor dysfunction were excluded.

### 2.12 Local retention assessment of PTA@Rop

FITC-labeled PTA@Rop was locally administered at the tumor-bearing femur. The fluorescence signal at the administration site was monitored at the indicated time points using an *in vivo* fluorescence imaging system. The fluorescence intensity was quantified using the same region of interest. Meanwhile, the retained Rop at the administration site was independently determined by HPLC. Briefly, the tissues around the administration site were collected, weighed, homogenized and extracted. After centrifugation, the Rop concentration in the supernatant was calculated according to the standard curve. These experiments were used to evaluate the local retention of PTA@Rop, but not to investigate the complete systemic mass balance or organ biodistribution.

### 2.13 Behavioral pain assessment

Behavioral assessments were performed double-blinded between 9:00 and 14:00. Mice were acclimated to the testing environment for at least 2 days before baseline measurements. Mechanical sensitivity was measured using von Frey filaments (0.04-2.0 g, Stoelting). Mice were placed on an elevated wire mesh platform, filaments were applied to the hind paw plantar surface, and the 50% withdrawal threshold was determined by the up-down method. Thermal sensitivity was evaluated with the Hargreaves test. Paw withdrawal latency to thermal irradiation was recorded (cutoff: 20 s). Locomotor function was assessed in an open field (45 × 45 cm). Movement trajectories were captured by an overhead camera and analyzed using SMART software to quantify total distance and average speed. Gait was analyzed using the CatWalk system, which records paw prints on an illuminated glass runway. Key parameters of paw print intensity, swing phase, stance phase, and stride length were quantified to assess pain-related limb dysfunction and treatment efficacy.

### 2.14 X-ray and Micro-CT bone imaging

Femurs from mice were scanned at 55 kVp using a VivaCT 80 scanner (Scanco Medical, Switzerland) to quantify bone volume fraction (BV/TV) and connectivity density (Conn.D.) in the distal femur. Bone destruction scores were evaluated from X-ray images captured with an X-Viewer XV-108 S analyzer.

### 2.15 Spinal cord histology

Lumbar spinal cords (L4–L6) were harvested after cardiac perfusion, cryoprotected in 30% sucrose, and sectioned at 20 μm on a cryostat. Sections were processed for Nissl staining or immunofluorescence using anti-NGF, anti-c-Fos, and anti-CGRP antibodies, and images were acquired with a confocal microscope. Representative RNA-seq targets were evaluated by RT-qPCR using spinal-cord RNA. Relative expression of *Tnf, Il1b, Il6, Ccl2,* and *Nfkbia* were normalized to Gapdh, with the primer sequences are provided in Supplementary Table S2.

### 2.16 Drug target prediction and bioinformatics analysis

The molecular mechanism underlying the neuroprotective effects of thioctic acid (TA) was investigated by predicting potential protein targets via the SwissTargetPrediction database (http://www.swisstargetprediction.ch/). Using the chemical structure of TA as a query and “*Mus musculus*” as the target species, candidates with a probability score >0 were selected for further analysis. Core signaling networks were identified through functional annotation and pathway enrichment using the KEGG database.

### 2.17 Statistics analysis

Statistical analyses were performed using GraphPad Prism 9.0 software. Data are presented as mean ± SD. Statistical significance was determined by one- or two-way ANOVA followed by Tukey's post-hoc test. *p < 0.05, **p < 0.01, ***p < 0.001.

## 3 Results and discussion

### 3.1 Ropivacaine provides transient analgesia but paradoxically aggravates spinal neuronal injury in BCP

Bone cancer pain (BCP) is a complex pathological state driven by malignant bone destruction and maladaptive central sensitization. To evaluate the therapeutic efficacy and potential limitations of clinical local anesthetics, we established a murine BCP model via intra-femoral inoculation [39] of Lewis lung cancer cells (Fig. 1A). Tumor-bearing mice developed progressive mechanical and thermal nociception [40], as reflected by decreased mechanical withdrawal threshold and thermal withdrawal latency (Fig. 1B, C, Fig. S1) [41]. Although ropivacaine (Rop) administration produced immediate analgesia, this anti-nociceptive effect was transient, with pain thresholds rapidly returning to baseline. Notably, X-ray and micro-CT analysis [42] further suggested that Rop failed to inhibit tumor-induced osteolysis (Fig. 1D, Fig. S2–S3). Severe cortical erosion and trabecular fractures were observed in Rop-treated mice, accompanied by a decrease in bone volume fraction (BV/TV) and reduced connectivity density (Conn. D). Consistently, TRAP staining revealed significant bone loss and extensive osteoclast activation, corroborating the imaging findings.

**Fig. 1:**
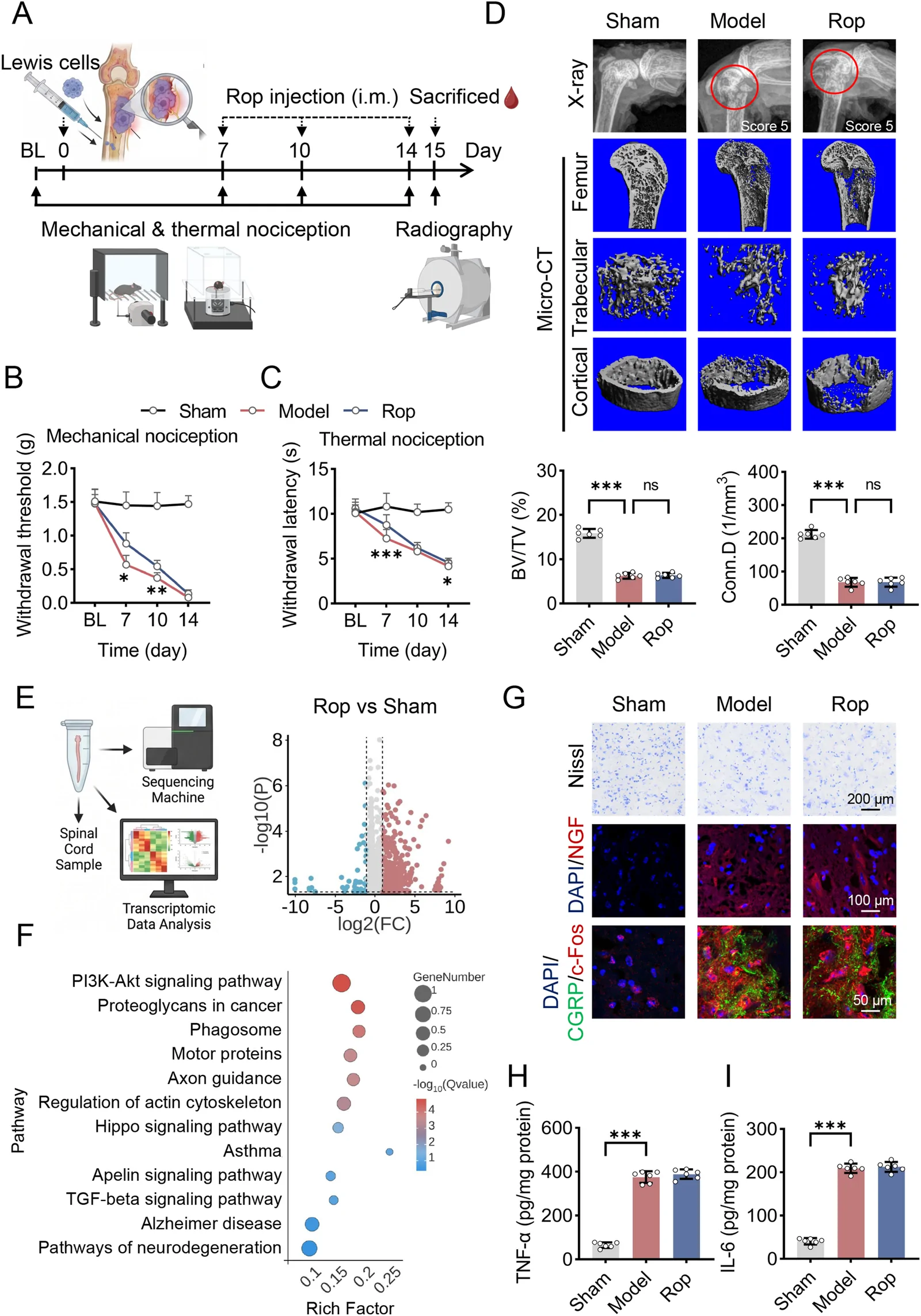
Ropivacaine exhibits transient analgesia and exacerbates spinal neuronal damage in BCP mice. (A) Schematic representation of the BCP model establishment via intra-femoral inoculation of Lewis lung cancer cells, the drug administration schedule, and experimental procedures. (B) Mechanical withdrawal threshold and (C) thermal withdrawal latency measured at the indicated time points. (D) Representative X-ray images (red circles indicate bone destruction; radiographic scores are labeled) and 3D micro-CT reconstructions (femur, trabecular, and cortical bone) of femurs. (E) Quantitative bone histomorphometry analysis of bone volume fraction (BV/TV) and connectivity density (Conn. D). (F) Schematic workflow of spinal cord transcriptomic analysis and volcano plot showing differentially expressed genes (DEGs) in the Rop vs sham. (G) KEGG pathway enrichment analysis of upregulated DEGs in the Rop vs sham comparison. (H) Representative images of Nissl staining, NGF immunofluorescence (NGF: red; DAPI: blue), and CGRP/c-Fos immunofluorescence (CGRP: green; c-Fos: red; DAPI: blue) in the spinal cord. Scale bar: 200 μm (Nissl), 100 μm (NGF), 50 μm (CGRP/c-Fos). (I, J) Levels of TNF-α (I) and IL-6 (J) in spinal cord tissue measured by ELISA. Data are presented as mean ± SD (n = 6). Statistical significance was determined by one- or two-way ANOVA followed by Tukey's post-hoc test. *p < 0.05, **p < 0.01, ***p < 0.001. (For interpretation of the references to colour in this figure legend, the reader is referred to the Web version of this article.)

More importantly, the effects of sustained Rop exposure on the spinal microenvironment were further explored. Transcriptomic analysis profiling with a volcano plot of spinal cord tissues revealed significant upregulation of gene clusters related to the PI3K-Akt, axon guidance, and TGF-β signaling pathways in Rop-treated BCP mice (Fig. 1E and F). Nissl staining showed neuronal morphological deterioration [43], including neuronal injury and reduced neuronal density in the spinal cord after Rop administration (Fig. 1G, Fig. S4). Immunofluorescence staining of the spinal cord visualized the expression of NGF, CGRP (green), and c-Fos (red) to evaluate pain-related signaling, neuronal morphology, and neuronal activation, respectively (Fig. S5–S6). The combined fluorescence data verified that Rop treatment disrupted normal neuronal morphology and aggravated abnormal pain-associated neuronal hyperexcitability. Additionally, ELISA assays suggested that Rop elevated the protein levels of the inflammatory cytokines TNF-α and IL-6 (Fig. 1H and I). Collectively, these data demonstrate that Rop provides transient analgesia but aggravates spinal neuronal injury and neuroinflammation, ultimately promoting central sensitization in BCP.

### 3.2 Integrative screening identifies thioctic acid (TA) as a potent neuroprotective countermeasure

Given the undesirable neuronal damage triggered by Rop during analgesic treatment, we sought to develop an optimized combination strategy incorporating neuroprotective agents to alleviate Rop-induced secondary neuronal injury. Thioctic acid (TA), a naturally occurring antioxidant, emerged as a promising candidate capable of reversing the pathological gene expression profile induced by Rop. To further explore its underlying function, we performed systematic drug target prediction using the SwissTargetPrediction database to screen potential target genes and downstream signaling pathways (Fig. 2A and B). Bioinformatic analysis revealed that TA predominantly downregulated inflammation-related pathways and the neuroactive ligand-receptor interaction pathway, which may contribute to mitigating Rop-induced spinal neuronal injury.

**Fig. 2:**
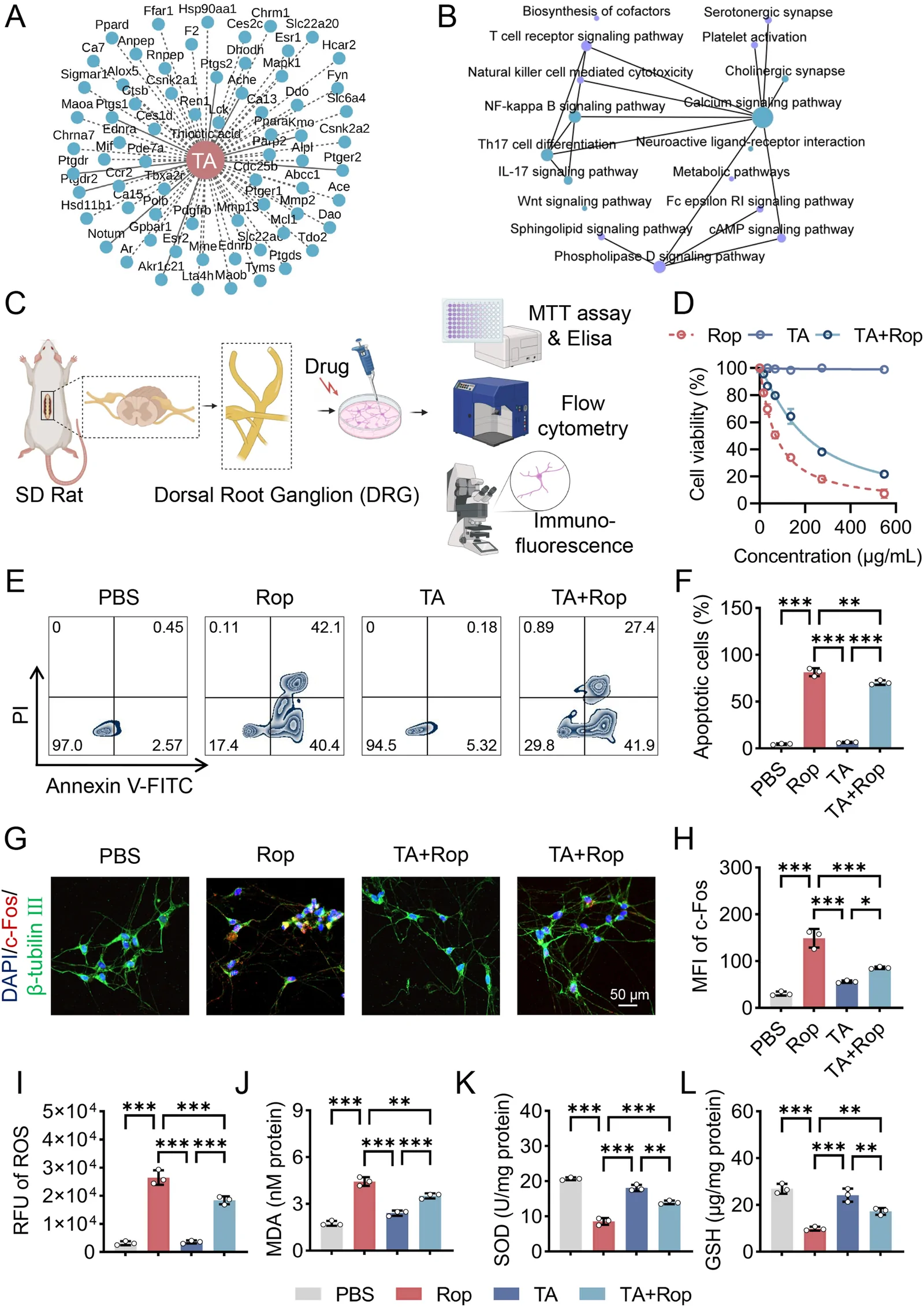
Thioctic acid (TA) counteracts ropivacaine (Rop)-induced neurotoxicity in primary rat dorsal root ganglion (DRG) neurons. (A, B) Drug-target prediction (A) and KEGG pathway enrichment analysis (B) of TA. (C) Schematic illustration of the *in vitro* experimental workflow using primary DRG neurons, including MTT, flow cytometry, and immunofluorescence assays. (D) Cell viability of DRG neurons treated with different concentrations of Rop, TA, or their combination (TA + Rop). (E, F) Representative flow cytometry plots (E) and quantification (F) of Annexin V/PI staining. (G, H) Representative immunofluorescence images (G) and mean fluorescence intensity (MFI) quantification (H) of c-Fos expression in DRG neurons (β-tubulin III: green; c-Fos: red; DAPI: blue). Scale bar: 50 μm. (I–L) Quantification of intracellular reactive oxygen species (ROS) (I), malondialdehyde (MDA) levels (J), superoxide dismutase (SOD) activity (K), and glutathione (GSH) content (L) in DRG neurons. Data are presented as mean ± SD (n = 3). Statistical significance was determined by one- or two-way ANOVA followed by Tukey's post-hoc test. *p < 0.05, **p < 0.01, ***p < 0.001. (For interpretation of the references to colour in this figure legend, the reader is referred to the Web version of this article.)

To verify the neuroprotective effect of TA, primary rat dorsal root ganglion (DRG) neurons were isolated and cultured for *in vitro* pharmacological validation (Fig. 2C). The neurotoxic potential of Rop toward sensory neurons was showed *in vitro*. DRG neurons exposed to Rop (0-600 μg/mL) exhibited a dose-dependent reduction in cell viability and a significant increase in the apoptotic population (Fig. 2D). As showed by flow cytometry, TA alone exhibited negligible cytotoxicity against DRG neurons, whereas TA supplementation efficiently ameliorated Rop-induced neuronal apoptosis. Functional validation showed that TA co-treatment significantly rescued DRG neurons from Rop-induced injury. TA administration markedly reduced the population of Annexin V/PI-positive apoptotic cells and restored metabolic activity (Fig. 2E and F).

Furthermore, TA effectively suppressed neuronal hyperexcitability, as evidenced by restored axonal morphology (β-tubulin III, green) and reduced c-Fos expression (red) in DRG neurons (Fig. 2G, H, Fig. S7). While the Rop group displayed prominent axonal disruption and excessive neuronal activation, these pathological changes were markedly attenuated by TA treatment. Mechanistically, TA exerted robust antioxidant effects by reversing Rop-induced redox imbalance, which significantly reduced intracellular reactive oxygen species (ROS) and malondialdehyde (MDA) levels, while restoring the activity of endogenous antioxidant systems, including superoxide dismutase (SOD) and glutathione (GSH) (Fig. 2I–L, Fig. S8). Collectively, these results demonstrate that TA acts as a potent neuroprotective agent to counteract Rop-induced neuronal apoptosis, oxidative stress and neuroinflammation, laying a solid foundation for the subsequent development of combination therapeutic strategies for BCP.

### 3.3 Engineering and characterization of reduction-responsive PTA@Rop nanoparticles for synergistic analgesia and neuroprotection

Guided by the potent neuroprotective efficacy of TA, we engineered a multifunctional nanoplatform to synchronize prolonged analgesia with neuronal preservation in the BCP microenvironment. Leveraging the 1,2-dithiolane ring structure of TA, we first induced its temperature-induced ring-opening polymerization to form polythioctic acid (PTA). Subsequently, PTA was conjugated with mPEG-NH_2_ and loaded with ropivacaine (Rop) to self-assemble into reduction-responsive PTA@Rop nanoparticles, which were designed to integrate prolonged analgesia with neuroprotective effects (Fig. 3A, Fig. S9).

**Fig. 3:**
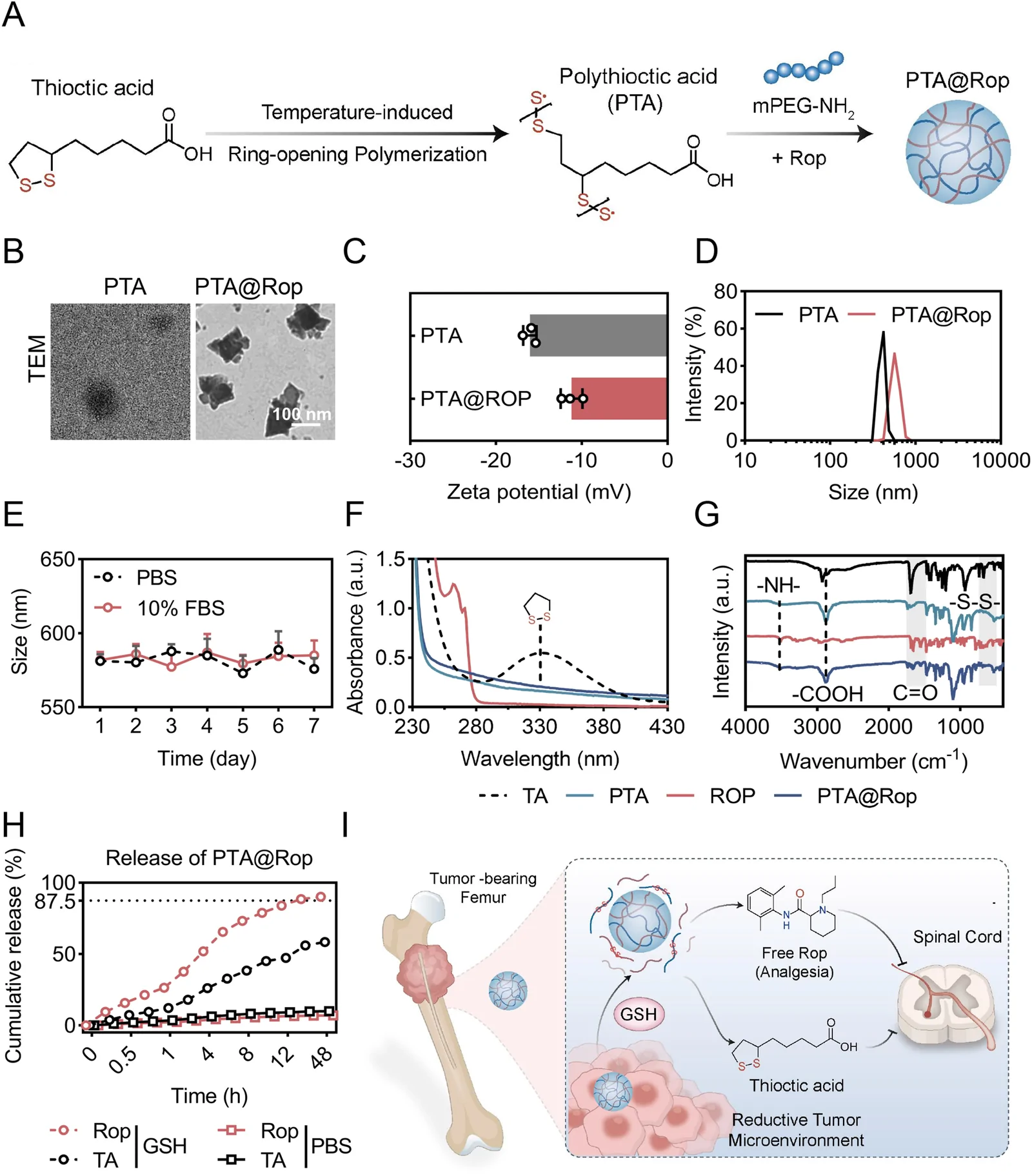
Fabrication and physicochemical characterization of the reduction-responsive PTA@Rop nanoplatform. (A) Schematic illustration of the synthesis route of PTA nanoparticles via ring-opening polymerization and mPEG-NH_2_ conjugation with Rop loading. (B) Representative TEM images of PTA and PTA@Rop nanoparticles. Scale bar: 100 nm. (C, D) Zeta potential (C) and hydrodynamic size distribution (D) of PTA and PTA@Rop. (E) Particle size stability of PTA@Rop over 7 days in PBS and 10% FBS. (F) UV-vis absorption spectra. (G) FTIR spectra of TA, PTA, Rop, and PTA@Rop, showing characteristic peaks for N–H, C=O, and S–S bonds. (H) *In vitro* cumulative release profiles of Rop and TA from PTA@Rop in PBS or GSH over 48 h. (I) Schematic diagram illustrating the reduction-responsive drug release mechanism of PTA@Rop in the tumor microenvironment, enabling synchronous release of Rop (analgesia) and TA (neuroprotection). Data are presented as mean ± SD.

Physicochemical characterization showed the formation of spherical PTA@Rop nanoparticles with hydrodynamic diameters of approximately 480-660 nm and a surface charge of approximately −13 mV (Fig. 3B–D). The particle size remained relatively stable during the 7-day incubation period (Fig. 3E), indicating good stability under the tested conditions. Such a stability profile might be attributed to the amphiphilic and dynamic exchange properties of PEG-conjugated PTA [44]. The UV-vis spectrum of PTA showed no characteristic dithiolane peak at 330 nm, confirming complete ring-opening polymerization of the TA monomer (Fig. 3F). Fourier-transform infrared (FTIR) spectroscopy showed characteristic absorption bands at approximately 3500 and 1680 cm^−1^, which were assigned to the N–H and C=O stretching vibrations of the amide group in Rop, respectively. The absorption bands at 500–550 cm^−1^ were attributed to the S–S stretching vibration in the PTA backbone (Fig. 3G). Gel permeation chromatography (GPC) analysis further showed that the synthesized PTA had a number-average molecular weight (M_n_) of 1406 Da and a dispersity (Đ) of 1.364 (Fig. S10). These results supported the successful synthesis of oligomeric PTA and confirmed its molecular-weight characteristics.

The *in vitro* release profiles of Rop and TA from PTA@Rop nanoparticles were evaluated in PBS and 10 mM GSH over 48 h (Fig. 3H). Across three independently prepared batches, the drug-loading content and encapsulation efficiency of Rop were 8.2 ± 1.1% and 72.0 ± 9.9%, respectively (Table S2). In the presence of 10 mM GSH, both Rop and TA showed sustained and time-dependent release, with Rop being released relatively faster than TA. In contrast, only negligible release of Rop and TA was observed in PBS over 48 h, indicating limited premature drug release under non-reducing conditions. The local retention of PTA@Rop was further evaluated after in situ administration at the tumor-bearing femur. IVIS imaging and HPLC analysis consistently showed that PTA@Rop remained at the administration site for a longer period than free Rop (Fig. S11), supporting its improved local retention. This effect may be attributed to the nanoparticle formulation and the limited premature release of Rop under non-reducing conditions. In contrast, PLGA@Rop, which showed particle size and PDI comparable to those of PTA@Rop, exhibited no obvious GSH-accelerated Rop release (Fig. S12). This comparison suggests that the accelerated release from PTA@Rop is mainly associated with the GSH-sensitive disulfide-rich PTA matrix, rather than being a general consequence of nanoparticle size or drug encapsulation. GSH-induced cleavage and reorganization of the PTA matrix may increase drug diffusion from the nanoparticles, thereby promoting Rop release under reducing conditions. These findings demonstrated that the disulfide-rich PTA matrix reduces premature drug release, improves local drug retention, and responds to a reducing environment to promote drug release. Such a reduction-responsive property may facilitate the sustained local release of Rop and TA in the BCP microenvironment (Fig. 3I). Nevertheless, the *in vitro* release and local-retention results do not directly demonstrate systemic bioavailability. Further pharmacokinetic and quantitative biodistribution studies are required to investigate the *in vivo* disposition of PTA@Rop.

Cytotoxicity tests in DRG, N_2_A, L929, and C2C12 cells revealed that Rop induced dose-dependent cytotoxicity, whereas the simple combination of TA and Rop provided only partial protective effects. In contrast, the engineered PTA@Rop nanoparticles showed acceptable cytocompatibility under the tested conditions, with cell viability remaining consistently above 85% even at 400 μg/mL, confirming their ability to mitigate Rop-induced toxicity (Fig. 4A–D). Consistent with these assays, flow cytometry showed that Rop induced severe apoptosis in DRG cells, which was partially attenuated in the TA + Rop group and markedly reduced in the PTA@Rop group (Fig. 4E and F). Immunofluorescence staining further suggested that Rop significantly upregulated the neuronal injury marker c-Fos, while both TA + Rop and PTA@Rop treatments mitigated this effect, with the nanoparticles showing the strongest protection (Fig. 4G, H and Fig. S13). Mechanistically, Rop triggered substantial oxidative stress, as evidenced by elevated ROS and MDA levels, along with decreased SOD and GSH activity (Fig. 4I–L and Fig. S14). While the simple combination of TA and Rop only partially improved these redox parameters, the engineered PTA@Rop nanoparticles effectively improved cellular redox homeostasis by suppressing elevated ROS and MDA levels and replenishing endogenous SOD and GSH reservoirs, demonstrating their superior ability to protect neurons from Rop-induced oxidative damage.

**Fig. 4:**
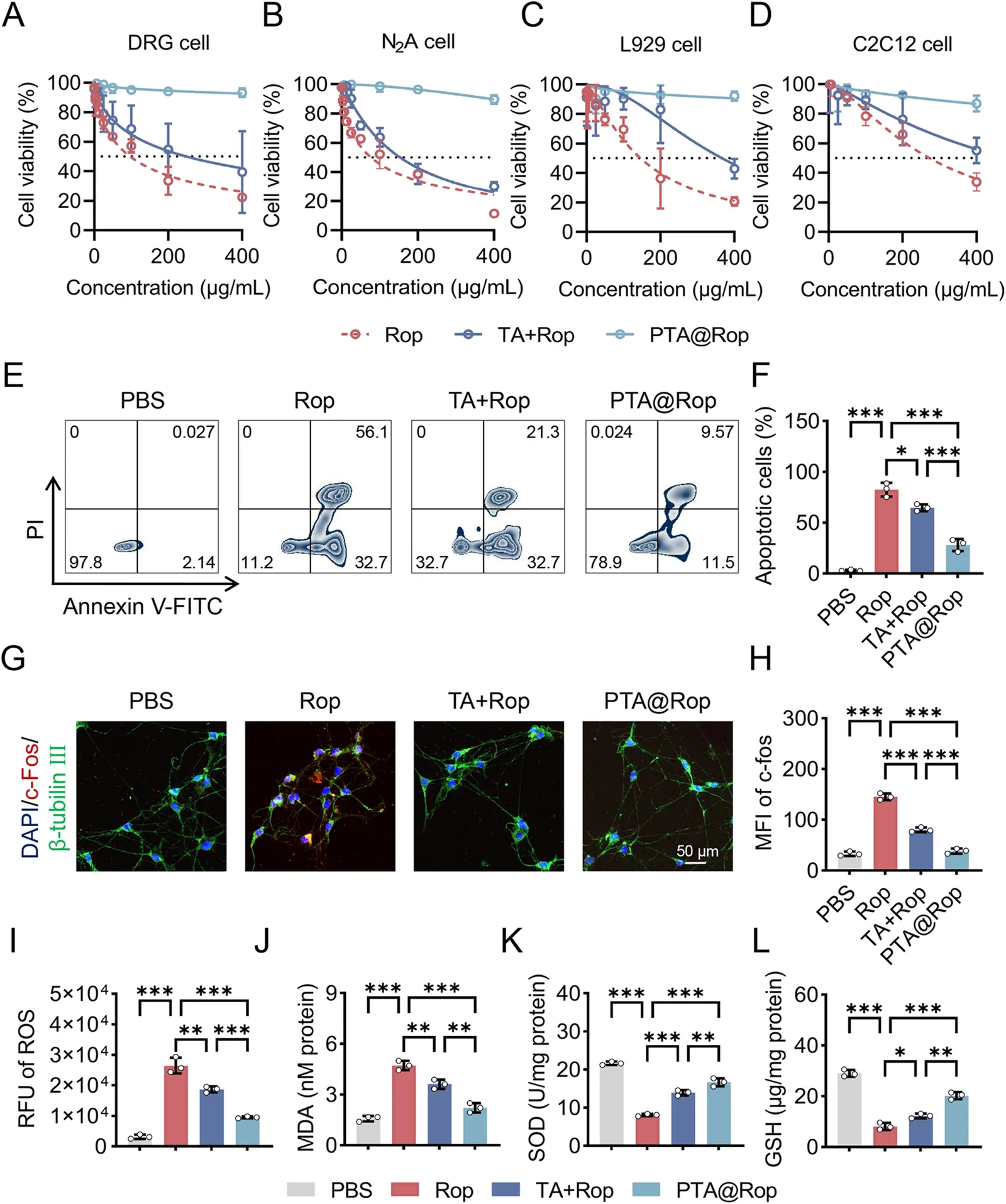
PTA@Rop nanoparticles attenuate ropivacaine-induced neurotoxicity and oxidative stress *in vitro*. (A–D) Cell viability of DRG (A), N_2_A (B), L929 (C), and C2C12 (D) cells after treatment with various concentrations. (E, F) Representative flow cytometry plots (E) and quantification (F) of Annexin V/PI. (G, H) Representative immunofluorescence images (G) and MFI quantification (H) of c-Fos expression in DRG neurons (β-tubulin III: green; c-Fos: red; DAPI: blue). Scale bar: 50 μm. (I–L) Quantification of intracellular ROS (I), MDA levels (J), SOD activity (K), and GSH content (L) in DRG neurons. Data are presented as mean ± SD (n = 3). Statistical significance was determined by one- or two-way ANOVA followed by Tukey's post-hoc test. *p < 0.05, **p < 0.01, ***p < 0.001. (For interpretation of the references to colour in this figure legend, the reader is referred to the Web version of this article.)

### 3.4 Sustained analgesia and preserved locomotor function by PTA@Rop in a mouse model of postoperative pain

To rigorously evaluate the translational potential and analgesic longevity of PTA@Rop nanoparticles, we employed a mouse plantar incision pain model (Fig. 5A and Fig. S15). The experimental timeline included baseline nociceptive testing (BL), drug administration immediately after surgery, and serial assessments of mechanical and thermal nociception at 0.5, 2, 4, 6, 8, 24, and 48 h post-incision. Open field tests were performed at 6 and 24 h to assess pain-related behavioral changes, followed by tissue collection at 48 h for further analysis.

**Fig. 5:**
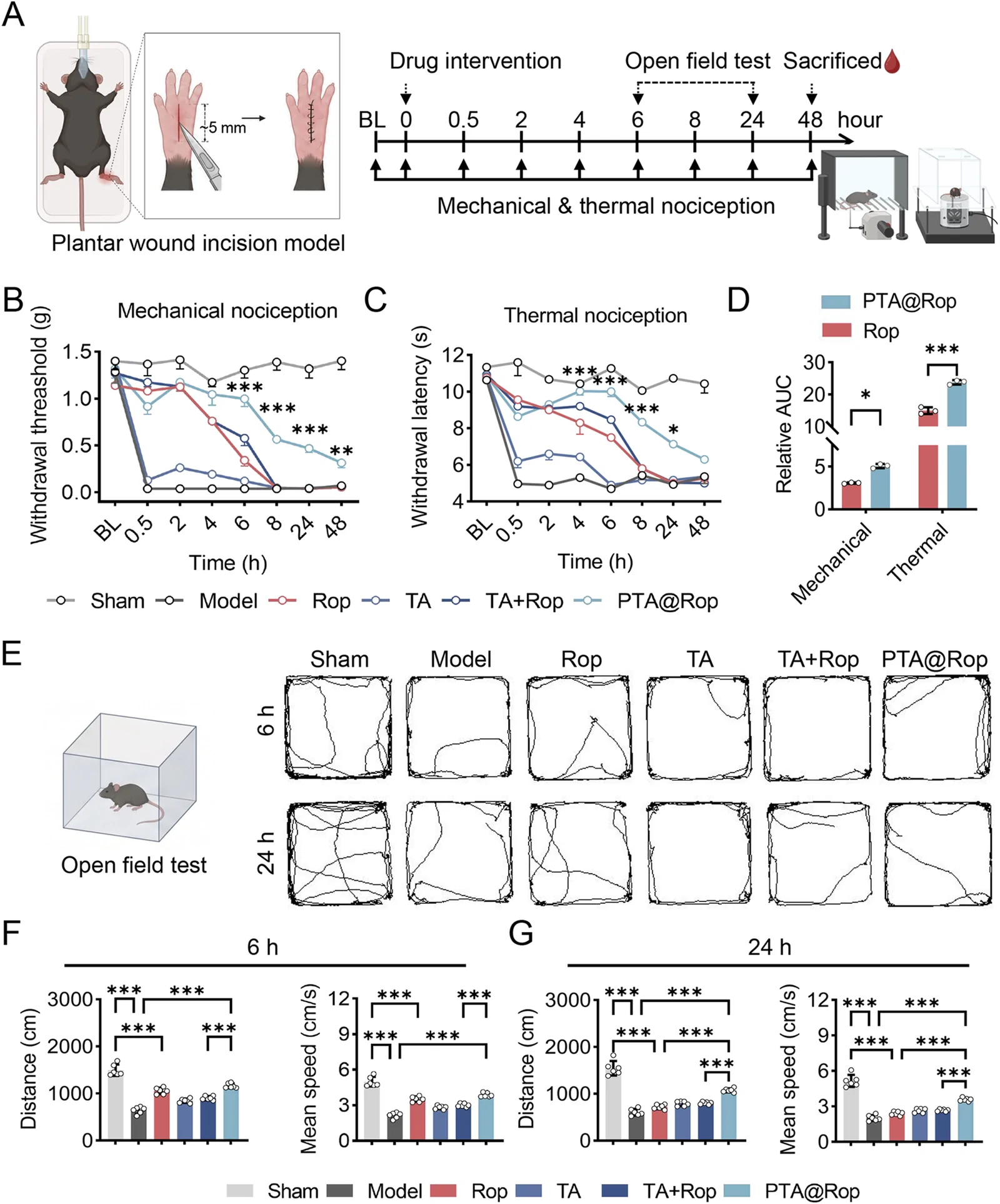
PTA@Rop provides prolonged analgesia and restores locomotor function in a mouse plantar incision pain model. (A) Schematic representation of the plantar incision pain model establishment, drug administration schedule, and experimental workflow. (B, C) Mechanical withdrawal threshold (B) and thermal withdrawal latency (C) measured at the indicated time points. (D) Relative area under the curve (AUC) analysis of mechanical and thermal nociceptive responses, quantifying the duration of analgesic effect. (E) Representative locomotor traces from the open field test at 6 h and 24 h post-incision. (F, G) Quantification of total travel distance and mean speed at 6 h (F) and 24 h (G) in the open field test. Data are presented as mean ± SD (n = 6–8). Statistical significance was determined by one- or two-way ANOVA followed by Tukey's post-hoc test. *p < 0.05, **p < 0.01, ***p < 0.001.

Behavioral assessments of mechanical and thermal nociception revealed distinct analgesic profiles across treatment groups (Fig. 5B and C). The model group exhibited rapid and sustained reduction in both mechanical withdrawal threshold and thermal withdrawal latency, indicating robust pain hypersensitivity. Free Rop produced only transient analgesia, mechanical thresholds peaked at 2 h but returned to baseline hyperalgesia by 6 h, while thermal withdrawal latency similarly declined after 4 h. TA + Rop combination provided modest improvements, extending analgesia slightly compared to free Rop. In contrast, PTA@Rop nanoparticles elicited prolonged and robust pain relief, maintaining elevated mechanical withdrawal thresholds for over 24 h and thermal withdrawal latencies up to 48 h. Quantification of the relative area under the curve (AUC) confirmed that PTA@Rop increased the duration of analgesia by 1.65-fold for mechanical nociception and 1.59-fold for thermal nociception compared to free Rop (Fig. 5D). The prolonged behavioral effect was consistent with the sustained local release of Rop from PTA@Rop, which might be useful to maintain an effective drug concentration at the administration site.

Consistent with the nociceptive data, open field tests at 6 and 24 h showed that the model group exhibited significantly reduced total travel distance and mean speed, indicative of pain-induced behavioral depression (Fig. 5E–G). Free Rop only partially improved these parameters at 6 h, with no sustained benefit at 24 h. In contrast, PTA@Rop significantly restored locomotor activity with total distance and mean speed markedly higher than those in the model, Rop, and TA + Rop groups. These findings confirm that PTA@Rop effectively alleviates pain-related functional impairment over the 24 h observation period. The consistent improvement in nociceptive and locomotor parameters suggests that the effect of PTA@Rop is not limited to a single behavioral endpoint.

### 3.5 Sustained analgesia and functional recovery by PTA@Rop in a mouse model of bone cancer pain

To further investigate the therapeutic efficacy of PTA@Rop under chronic pain conditions, a bone cancer pain (BCP) model was established by intra-femoral inoculation of Lewis lung cancer cells (Fig. 6A, Fig. S16). Tumor-bearing mice showed sustained decreases in mechanical withdrawal threshold and thermal withdrawal latency, indicating the development of mechanical and thermal hypersensitivity (Fig. 6B and C). Free Rop produced only transient relief, whereas PTA@Rop maintained higher mechanical and thermal thresholds throughout the observation period. Similar analgesic effects were also observed in the 4T1-derived BCP model (Fig. S17), supporting the reproducibility of the therapeutic effect in a second tumor-associated pain model. The prolonged analgesia of PTA@Rop may be mainly associated with the sustained local release of Rop, while the antioxidant and neuroprotective activities of TA may additionally contribute to the attenuation of pain sensitization.

**Fig. 6:**
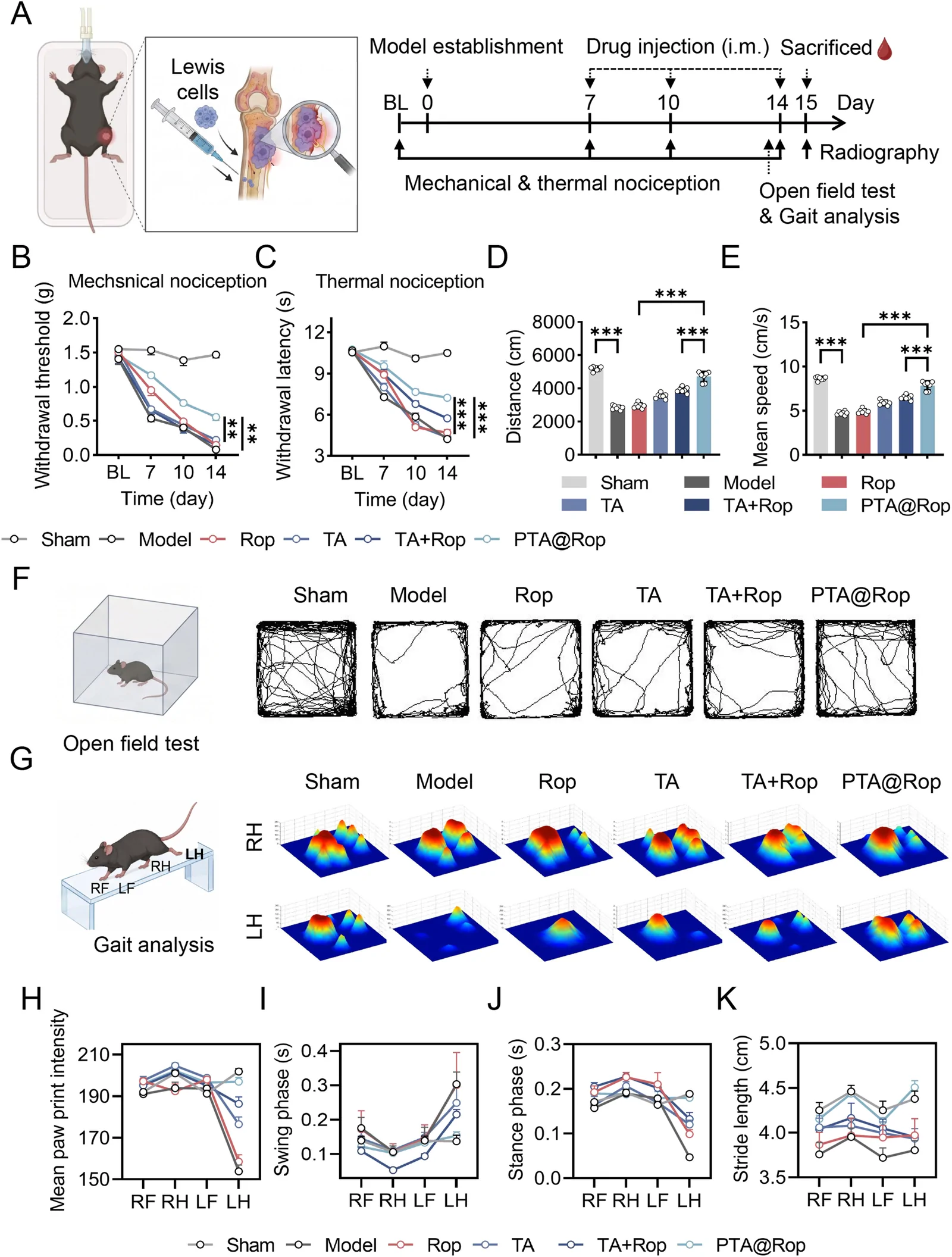
PTA@Rop provides sustained analgesia and restores locomotor function in the BCP model. (A) Schematic representation of the BCP model establishment via intra-femoral inoculation of Lewis cells, drug administration schedule, and experimental workflow. (B, C) Mechanical withdrawal threshold (B) and thermal withdrawal latency (C) measured at the indicated time points. (D, E) Quantification of total travel distance (D) and mean speed (E) in the open field test at day 14 post-inoculation. (F) Representative locomotor traces from the open field test at day 14. (G) Schematic diagram of gait analysis and representative 3D paw print intensity maps for each group. (H–K) Quantitative gait analysis, including mean paw print intensity (H), swing phase (I), stance phase (J), and stride length (K). Data are presented as mean ± SD (n = 6). Statistical significance was determined by one- or two-way ANOVA followed by Tukey's post-hoc test. *p < 0.05, **p < 0.01, ***p < 0.001.

The improvement in nociceptive thresholds was accompanied by better locomotor and gait performance. In the open field test, PTA@Rop-treated mice exhibited greater total travel distance and mean speed than mice in the model, Rop, and TA + Rop groups at day 14 (Fig. 6D–F). Gait analysis further showed improved paw-print intensity, stance phase, swing phase, and stride length in the affected hind limb (Fig. 6G–K and Fig. S18–S19). The consistent changes across nociceptive, locomotor, and gait parameters demonstrated that PTA@Rop not only produced sustained analgesia but also improved pain-related functional performance in BCP mice.

### 3.6 Integrated therapeutic effects of PTA@Rop on bone structure, neuroinflammation, and redox homeostasis in BCP

Given the intricate interplay between peripheral bone destruction and central sensitization in BCP, we evaluated the multi-organ protective capacity of PTA@Rop. X-ray imaging and 3D micro-CT reconstructions, complemented by H&E and TRAP staining, revealed severe cortical erosion and trabecular fractures in BCP mice (Fig. 7A–C,Fig. S20–S22). Notably, PTA@Rop-treated mice maintained relatively intact bone architecture, with quantitative histomorphometry showing significantly higher bone volume fraction (BV/TV) and connectivity density (Conn. D) compared to model, Rop, or TA + Rop groups. This suggests that sustained release of TA from the nanoparticles may play a secondary role in preserving bone integrity within the tumor microenvironment.

**Fig. 7:**
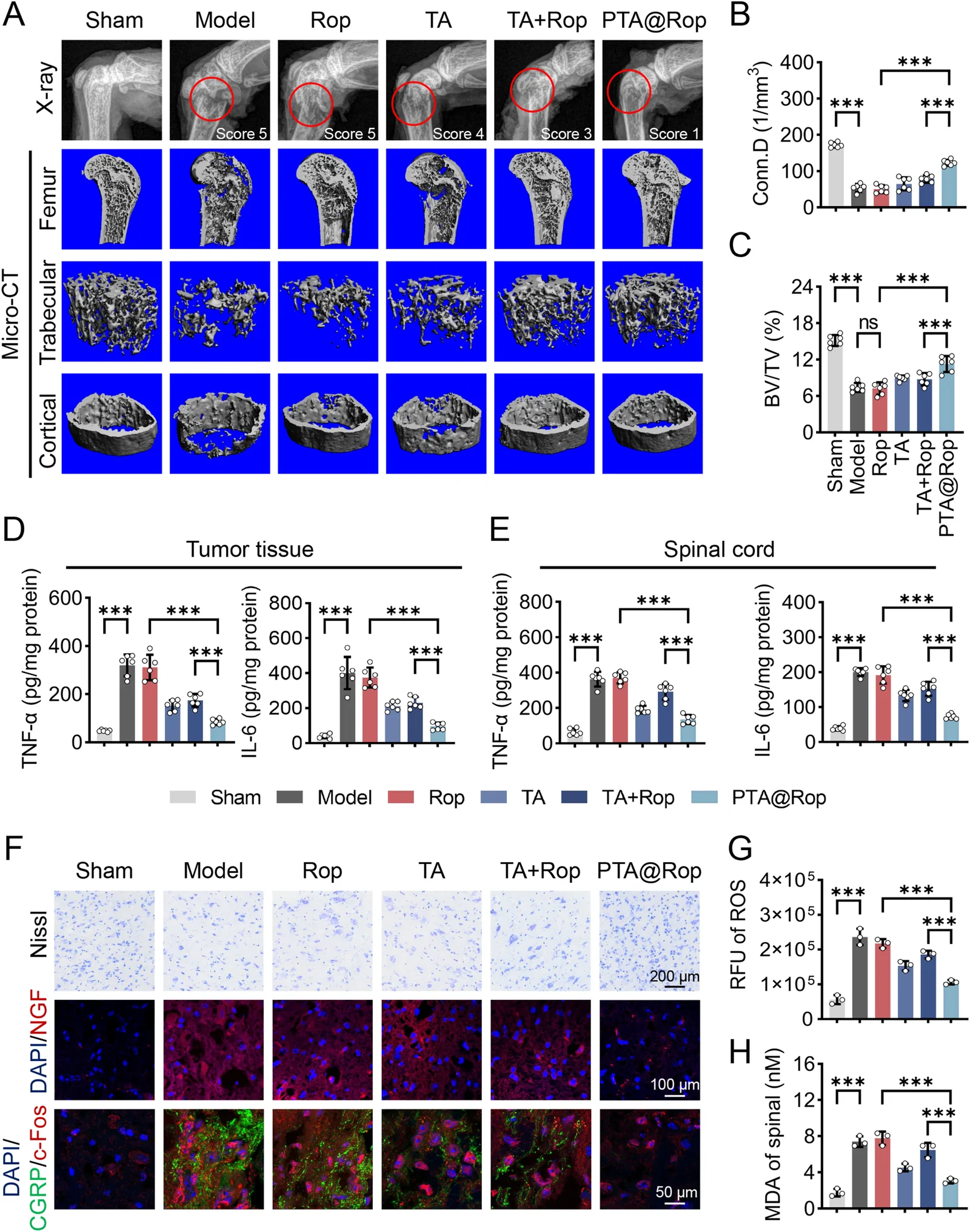
PTA@Rop preserves bone integrity and mitigates spinal inflammation and oxidative stress in BCP mice. (A) Representative X-ray images (red circles indicate bone destruction, radiographic scores are labeled) and 3D micro-CT reconstructions (femur, trabecular, and cortical bone) of femurs. (B, C) Quantitative bone histomorphometry analysis of Conn. D (B) and BV/TV (C). (D, E) Quantification of TNF-α and IL-6 levels in tumor tissue (D) and spinal cord (E). (F) Representative Nissl staining, NGF immunofluorescence, and CGRP/c-Fos immunofluorescence images of the spinal cord. Scale bars: 200 μm (Nissl staining), 100 μm (NGF), 50 μm (CGRP/c-Fos). (G, H) Quantification of intracellular ROS (G) and MDA levels (H) in spinal cord tissue. Data are presented as mean ± SD (n = 3-6). Statistical significance was determined by one- or two-way ANOVA followed by Tukey's post-hoc test. *p < 0.05, **p < 0.01, ***p < 0.001. (For interpretation of the references to colour in this figure legend, the reader is referred to the Web version of this article.)

We further interrogated the inflammatory and oxidative landscape at both the tumor site and the spinal cord to uncover the mechanisms underlying the efficacy of PTA@Rop. ELISA assays suggested that PTA@Rop treatment significantly suppressed the elevation of TNF-α and IL-6 in both tumor tissue and the spinal cord, whereas free Rop alone failed to provide such modulation (Fig. 7D and E). In the spinal cord, PTA@Rop effectively attenuated central sensitization, as evidenced by a marked reduction in neuronal activation markers c-Fos and CGRP, as well as the normalization of NGF expression (Fig. 7F and Fig. S23–S24). Nissl staining further shows that PTA@Rop alleviated the extensive neuronal damage and loss in the spinal dorsal horn of BCP mice (Fig. 7F).

Mechanistically, the neuroprotective advantage of PTA@Rop was reinforced by its ability to restore redox homeostasis. The nanoplatform markedly decreased intracellular ROS levels and MDA in spinal tissues, effectively reversing the oxidative stress induced by the tumor and exacerbated by Rop (Fig. 7G and H). Taken together, the coordinated improvements in bone structure, inflammatory signaling, neuronal activation, and oxidative stress demonstrated that the therapeutic effect of PTA@Rop involves both peripheral and central mechanisms. Sustained Rop release may contribute to prolonged nociceptive blockade, whereas the antioxidant activity of TA may help alleviate oxidative stress and neuroinflammation. However, the relative contribution of these two components required further investigation.

### 3.7 Transcriptomic profiling reveals that PTA@Rop reshapes the spinal transcriptome to suppress neuroinflammatory and pro-nociceptive pathways

To gain comprehensive molecular insights into the systemic neuroprotective effects of PTA@Rop, we performed RNA transcriptomics on spinal cord tissues. Differential expression analysis revealed profound transcriptomic reshaping in PTA@Rop-treated mice. Comparing the PTA@Rop group to the model group, we identified 21 significantly upregulated genes and 2326 downregulated genes. Comparing PTA@Rop to Rop, we found 41 upregulated and 2743 downregulated genes (Fig. 8A and B). The agreement between transcriptomic analysis and RT-qPCR validation supports the involvement of inflammatory signaling in the therapeutic response to PTA@Rop. A Venn diagram identified 953 core genes commonly modulated by PTA@Rop in both comparisons (Fig. 8C). KEGG pathway enrichment analysis of these core genes highlighted key pathways linked to BCP pathogenesis, including cytokine-cytokine receptor interaction, axon guidance, osteoclast differentiation, and NF-κB signaling.

**Fig. 8:**
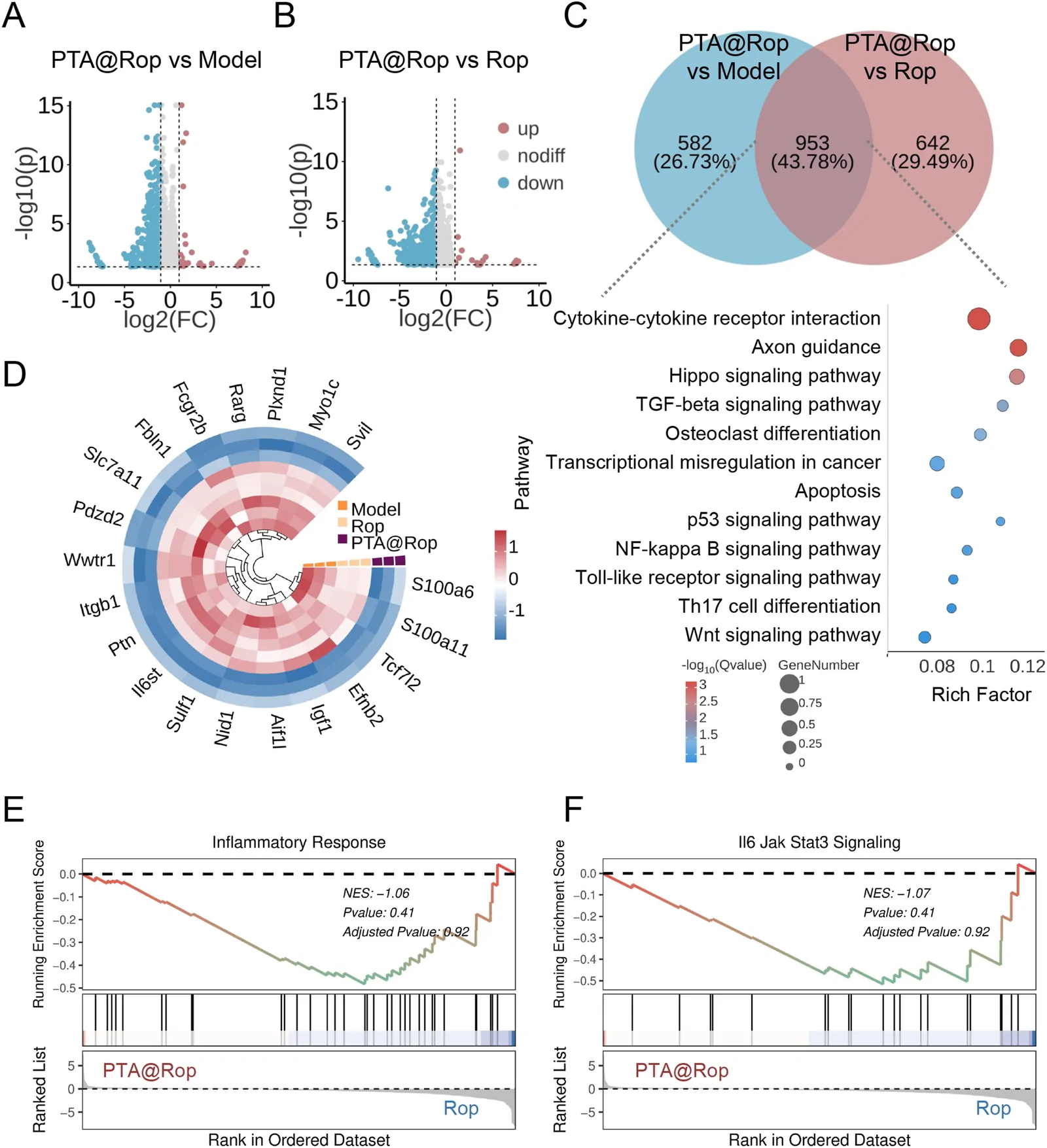
Transcriptomic analysis reveals that PTA@Rop reverses neuroinflammatory and pro-nociceptive signatures in the spinal cord of BCP mice. (A, B) Volcano plots showing DEGs in the spinal cord for PTA@Rop vs Model (A) and PTA@Rop vs Rop (B). Red dots represent upregulated genes, blue dots represent downregulated genes, and gray dots indicate no significant change. (C) Venn diagram illustrating the overlap of core DEGs modulated by PTA@Rop in both comparisons, with KEGG pathway enrichment analysis of the core gene sets. (D) Circular hierarchical clustering heatmap of representative DEGs. (E, F) Gene Set Enrichment Analysis (GSEA) showing the suppression of inflammatory response (E) and Il6-Jak-Stat3 signaling (F) pathways in the PTA@Rop vs Rop group comparison. (For interpretation of the references to colour in this figure legend, the reader is referred to the Web version of this article.)

A hierarchical clustering heatmap of representative differentially expressed genes (DEGs) showed clear segregation among the different groups. PTA@Rop treatment reversed the pro-inflammatory and pro-nociceptive gene expression profiles observed in the model and Rop groups (Fig. 8D). Gene Set Enrichment Analysis (GSEA) further showed that the inflammatory response and IL6-Jak-Stat3 signaling pathways were significantly suppressed in the PTA@Rop group (Fig. 8E and F). To further validate the RNA-seq results, the mRNA levels of five representative inflammation-related genes in the spinal cord were detected by RT-qPCR. In parallel to transcriptomic findings, Rop treatment increased the expression of *Tnf*, *Il1b*, *Il6*, *Ccl2*, and *Nfkbia*, whereas these changes were significantly reduced after PTA@Rop treatment (Fig. S25, Table S3).

### 3.8 Biocompatibility and systemic safety of PTA@Rop

A systematic biosafety evaluation is important for the potential clinical application of therapeutic nanoplatforms. H&E staining showed no obvious morphological damage, inflammatory infiltration, or pathological changes in the heart, liver, spleen, lung, and kidney of mice treated with PBS, free Rop, TA + Rop, or PTA@Rop (Fig. 9A). Serum levels of the hepatic function markers ALT and AST and the renal function indicators CREA and BUN showed no significant treatment-related changes, supporting the absence of detectable hepatic or renal injury under the tested conditions (Fig. 9B). During the 14-day repeated-dose study, PTA@Rop-treated mice showed stable body-weight changes and no significant differences in the organ coefficients of the heart, liver, spleen, lung, and kidney compared with PBS-treated mice (Fig. S26). Furthermore, the *in vitro* hemolysis assay showed negligible erythrocyte lysis at PTA@Rop concentrations of up to 10 mg/mL (Fig. 9C). Collectively, no obvious tissue injury or systemic toxicity was detected under the tested dose and observation period, supporting the preliminary biocompatibility of PTA@Rop nanoparticles.

**Fig. 9:**
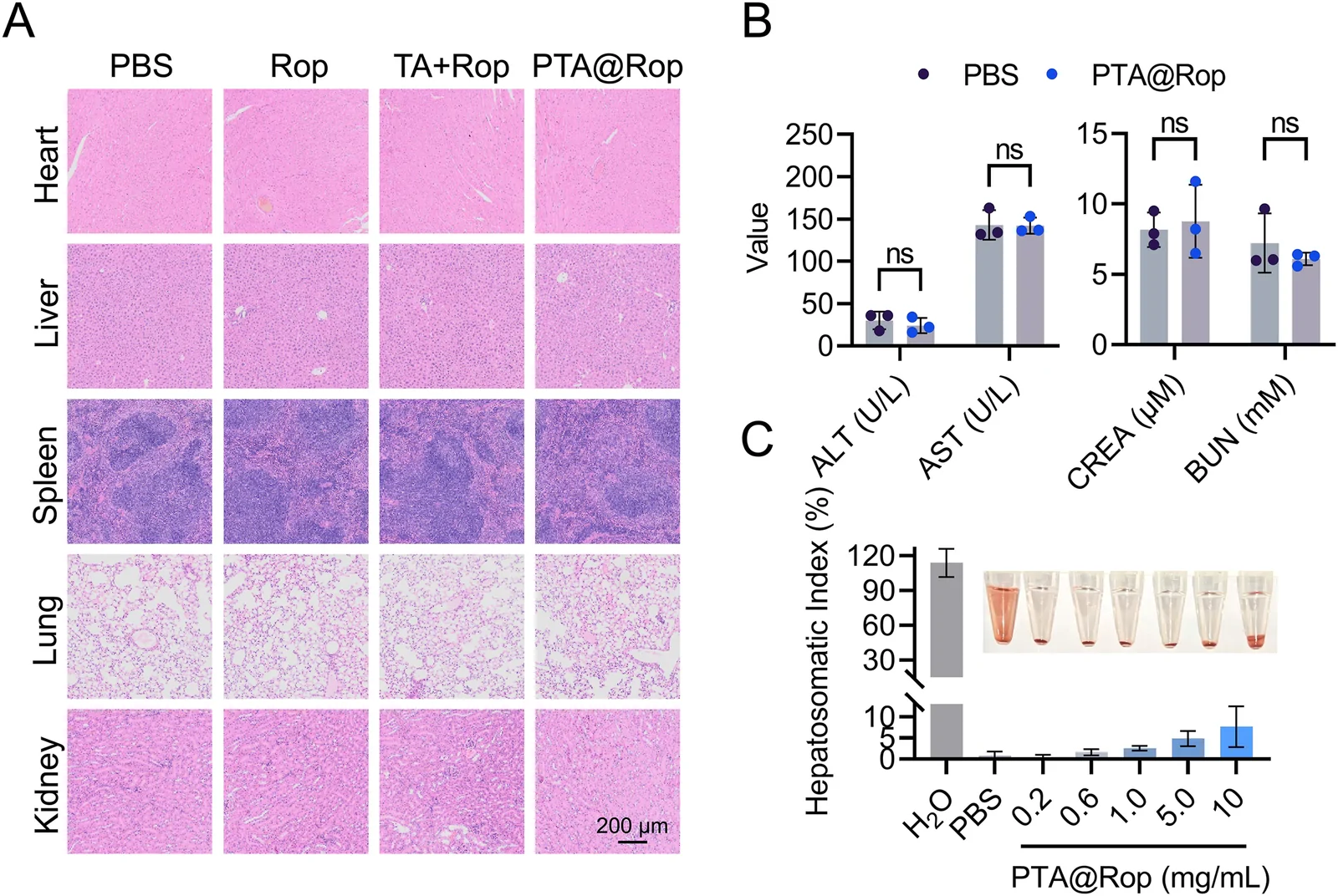
Biocompatibility and systemic safety evaluation of PTA@Rop. (A) H&E staining of major organs from mice treated with PBS, Rop, TA + Rop, or PTA@Rop. No obvious pathological changes were observed. Scale bars: 200 μm. (B) Serum biochemical analysis of hepatic (ALT, AST) and renal (CREA, BUN) function markers in different groups. (C) Hemolysis assay of PTA@Rop at various concentrations, showing negligible hemolytic activity. Data are presented as mean ± SD (n = 3).

## 4 Conclusion

Our study constructed a multifunctional tumor microenvironment-responsive nanoplatform, termed PTA@Rop to address the short analgesic duration and Rop-associated neuronal injury in this work. Using the intrinsic strained 1,2-dithiolane ring structure of thioctic acid (TA), we synthesized polythioctic acid (PTA) via a catalyst-free thermal ring-opening polymerization, followed by self-assembly and efficient encapsulation of Rop. Herein, we systematically confirmed that free Rop only produces transient pain relief and aggravates spinal neuronal damage, oxidative stress and neuroinflammation, thereby deteriorating central sensitization. In contrast, the engineered PTA@Rop nanoparticles have reduction-sensitive disulfide crosslinks, enabling reduction-responsive drug release underreducing conditions. The synchronously released Rop provides rapid nociceptive blockade for immediate analgesia, while the regenerated bioactive TA monomers effectively scavenge excess ROS and suppress neuroinflammatory cascades.

*In vitro* experiments and *in vivo* behavioral experiments verified the superior therapeutic performance of PTA@Rop. Compared with free Rop, this nanoplatform prolonged analgesic duration by 1.6-fold, and effectively restored gait function and weight-bearing capacity in BCP mice without detectable behavioral impairment. Mechanistically, transcriptomic sequencing showed that PTA@Rop profoundly reshapes the pathological spinal microenvironment. It prominently downregulates neuroinflammatory cytokines and nociceptive mediators, while also inhibiting multiple pain-related signaling pathways including cytokine-cytokine receptor interaction, axon guidance, and NF-κB signaling. These effects collectively alleviate peripheral bone destruction and reduce markers of spinal central sensitization. Furthermore, preliminary biosafety evaluation showed no obvious treatment-related toxicity under the tested conditions.

In summary, this work proposes an integrated biomaterial strategy to break the therapeutic bottleneck of traditional local anesthetics. The dual-delivery PTA@Rop nanoparticles achieve combined analgesic and neuroprotective effects, supporting further preclinical evaluation for prolonged analgesia in metastatic BCP. More importantly, our study establishes a feasible paradigm for the rational design of reduction-responsive and neuroprotective drug delivery systems, offering new insights for the optimization and further developing pain therapeutic regimens. Nevertheless, more studies on pharmacokinetics, quantitative biodistribution and long-term safety are needed to confirm the translational potential of PTA@Rop. Apart from redox-responsive strategies, mechanically activated nanosystems can also remodel tumor microenvironment for cancer treatment, such as mechanically regulated nanozymes and mechanoluminescence-based theranostic platforms [45,46]. Introducing physical-response modules into pain-targeted nanomedicines may provide new ideas for BCP therapy.

## CRediT authorship contribution statement

**Ziping Wu:** Writing – original draft, Project administration, Investigation, Data curation. **Yucen Zhang:** Writing – original draft, Project administration, Investigation, Data curation. **Yan Guo:** Software, Methodology, Formal analysis. **Yueying Wang:** Software, Methodology, Investigation. **Zhen Wang:** Resources, Investigation. **Zhuo Yang:** Resources, Investigation. **Dongmei Guo:** Validation, Formal analysis. **Chao Xu:** Validation, Formal analysis. **Boyuan Gu:** Visualization, Data curation. **Haoyu Zheng:** Validation, Data curation. **Yali Yan:** Supervision, Methodology. **Ming Zhang:** Writing – review & editing, Resources, Investigation, Formal analysis. **Dan Shao:** Writing – review & editing, Resources, Methodology, Investigation. **Li Chen:** Writing – review & editing, Resources, Methodology, Investigation. **Aitao Wang:** Writing – review & editing, Resources, Investigation, Funding acquisition.

## Declaration of generative AI and AI-assisted technologies in the manuscript preparation process

During manuscript preparation, ChatGPT was used for language polishing, grammar revision and logical expression optimization. The author(s) reviewed and edited the content as needed and take full responsibility for the published article.

## Declaration of competing interests

The authors declare that they have no known competing financial interests or personal relationships that could have appeared to influence the work reported in this paper.

## Data Availability

Data will be made available on request.
