# Supplementary material for: Bioactive polythioctic acid-engineered ropivacaine alleviates bone cancer pain through prolonged analgesia and multi-functional neuroprotection
Source: Mater Today Bio. 2026 Sep 18;41:103693. doi: 10.1016/j.mtbio.2026.103693 (PMC13631621; doi:10.1016/j.mtbio.2026.103693)
Supplement: Supplementary file 1 [file mmc1.docx]

**Bioactive Polythioctic Acid-Engineered Ropivacaine Alleviates Bone Cancer Pain Through Prolonged Analgesia and** **Multi-functional Neuroprotection**

*Ziping Wu**^a^, Yucen Zhang^b,^ ^d^, Yan Guo ^b, d^, Yueying Wang^e^, Zhen Wang^a^,* *Zhuo Yang^a^, Dongmei Guo^f^, Chao Xu^f^, Boyuan Gu^b^, Haoyu Zheng^b^, Yali* *Yan*^d^*, Ming Zhang^a^, Dan Shao*^,e^, Li Chen*^,^**^a^, and Aitao* *Wang*^,b,c,d^*

^a^ College of Basic Medical Sciences, the Medical Basic Research Innovation Center of Airway Disease in North China, Key Laboratory of Pathobiology, Ministry of Education, Jilin University, Changchun, 130021, China

^b^ Hohhot First Hospital, Hohhot, 010030, Inner Mongolia, China

^c^ Hohhot Stomatological Hospital, Inner Mongolia Autonomous Region Stomatological Hospital, Hohhot 010010, Inner Mongolia, China

^d^ Department of Anesthesiology, The Fifth Clinical Medical College, Inner Mongolia Medical University, Hohhot 010030, Inner Mongolia, China

^e^ State Key Laboratory of Oral Diseases and National Clinical Research Center for Oral Diseases and Department of Oral and Maxillofacial Surgery, West China Hospital of Stomatology, Sichuan University, Chengdu, Sichuan 610041, China

^f^ Department of Anesthesiology, Inner Mongolia People’s Hospital, Hohhot, Inner Mongolia, 010017, People’s Republic of China

* Corresponding author

E–mail addresses: Aitao Wang (tjxhmzk@163.com); Dan Shao (stanauagate@outlook.com); Li Chen (chenl@jlu.edu.cn);

**1. Experimental Procedures**

**1.1 Bone Histology and Histopathological Staining**

Femur samples were harvested and immediately fixed in paraformaldehyde (PFA) for 48 h. To facilitate sectioning, the samples were decalcified in a 10% ethylenediaminetetraacetic acid (EDTA, pH 7.4) solution at room temperature for 10 days, with the decalcifying medium refreshed every 48 h. Following decalcification, the tissues were dehydrated through a graded series of ethanol, cleared in xylene, and embedded in paraffin wax. Serial sections with a thickness of 5 μm were prepared using a rotary microtome. General tissue morphology and tumor cell infiltration were evaluated via H&E staining. To assess bone resorption activity, Tartrate-Resistant Acid Phosphatase (Trap) staining was performed using a commercial kit according to the manufacturer’s instructions. Mature osteoclasts were identified and quantified as TRAP-positive cells containing three or more nuclei located directly on the bone surface. Additionally, major systemic organs, including the heart, liver, spleen, lungs, and kidneys were harvested and processed using identical H&E staining protocols to evaluate the systemic biosafety of the administered formulations.

## **1.2 Radiographic Bone Destruction Scoring Method**

Femoral radiographic examination was performed using Faxitron Multi Focus X-ray system. To quantify tumor-induced bone deterioration, each femur was equally segmented into distal and proximal regions. Two independent investigators blinded to the experimental grouping scored every region according to a validated radiographic scoring system. Briefly, the scoring scale ranged from 0 to 5 based on the severity of bone resorption, medullary loss, cortical erosion, and bone fracture. The final bone destruction score of each femur was defined as the average score of the distal and proximal segments. The detailed scoring standards are listed in **Table S1**.

**1.3 Detection of Intracellular Oxidative Stress**

Intracellular redox homeostasis in primary DRG neurons was evaluated using commercial kits (Nanjing Jiancheng Bioengineering Institute, China). To quantify ROS generation, cells were incubated with 10 μM DCFH-DA for 30 min at 37 °C in the dark. Fluorescence intensity was then measured via laser scanning confocal microscope or a microplate reader (Ex/Em = 488/525 nm). For biochemical analysis, cells were lysed and centrifuged (12,000 rpm, 10 min, 4°C) to collect the supernatant. MDA content, SOD activity, and GSH levels were determined according to the manufacturer’s instructions. All results were normalized to total protein concentration via the BCA assay.

**1.4 Immunofluorescence Staining**

For cell culture, DRG neurons were fixed with 4% Perfluoroalkoxy for 15 min. For tissue sections, frozen spinal cord segments (L4-L6) were cut into 20 μm sections. Samples were permeabilized with 0.3% Triton X-100 for 15 min and blocked with 5% goat serum in PBS for 1 h. Primary antibodies against β-tubulin III (1:500), c-FOS (1:200), NGF (1:200), and CGRP (1:200) were applied overnight at 4°C. After washing with PBS, sections were incubated with Alexa Fluor-conjugated secondary antibodies (1:500) for 1 h at room temperature. Nuclei were counterstained with DAPI (1 μg/mL). Images were acquired using a laser scanning confocal microscope and analyzed using ImageJ software.

**1.5 Nissl Staining**

Frozen spinal cord sections were mounted on glass slides and immersed in Nissl staining solution for 10 min according to the manufacturer’s instructions. The sections were then rinsed in distilled water, dehydrated through 95% and 100% ethanol, cleared in xylene, and coverslipped. The integrity of neuronal structures, including the presence of Nissl bodies and the degree of neuronal pyknosis in the spinal dorsal horn, was examined under a light microscope.

**1.6 ELISA Assay**

Tissues from the tumor site and spinal cord were homogenized in RIPA lysis buffer containing protease inhibitors. The homogenates were centrifuged at 12,000 g for 15 min at 4°C. The concentrations of TNF-α and IL-6 in the supernatants were measured using specific ELISA kits (BioLegend) following the manufacturer's protocols. The optical density (OD) was measured at 450 nm using a microplate reader.

**1.7 Transcriptomic RNA-seq Analysis**

Spinal cord tissues were collected and total RNA was extracted using Trizol reagent. RNA quality was assessed using an Agilent 2100 Bioanalyzer. cDNA libraries were constructed and sequenced on an Illumina Novaseq 6000 platform. Clean reads were mapped to the mouse reference genome for data analysis. Differentially expressed genes were identified with criteria of | log_2_(Fold Change) | > 1 and adjusted P-value < 0.05. Functional annotation was performed via Kyoto Encyclopedia of Genes and Genomes (KEGG) and Gene Ontology (GO). Gene Set Enrichment Analysis (GSEA) was utilized to identify significantly enriched biological pathways.

**1.8 *In Vivo* Biosafety Evaluation**

In addition to histological analysis of the major organs, blood samples were collected from the retro-orbital venous plexus of mice. Serum was obtained by centrifugation at 3000 rpm for 10 min at 4°C and stored for biochemical analysis. Hepatic function was assessed by measuring the level of alanine aminotransferase (ALT) and aspartate aminotransferase (AST), while renal function was assessed by determining creatinine (CREA) and blood urea nitrogen (BUN) concentrations. For the hemolysis assay, PTA@Rop nanogels at various concentrations were incubated with 2% (v/v) red blood cell suspension for 2 h at 37 °C. Following incubation, the mixture was centrifuged, and the absorbance of the supernatant was recorded at 540 nm to calculate the hemolysis percentage.

**1.9 Dosage determination and experimental grouping**

For in *vitro* cellular experiments, the drug dosages were 50 μg/mL for Rop and 30 μg/mL for TA. For combination therapy (TA + Rop) group, the two drugs were mixed at their respective individual concentrations. For PTA@Rop, a concentration of 50 μg/mL was selected, corresponding to the Rop concentration used.

For *in vivo* animal experiments, the reference dose of Rop was set at 5 mg/kg. Based on the Rop loading content of the carrier, the administered dose of PTA@Rop nanogels was calculated to be 61 mg/kg. The corresponding dose of TA was 23.5 mg/kg. For the combination therapy (TA + Rop), the total dose consisted of 5 mg/kg Rop and 23.5 mg/kg TA.

**Figure S1.** Representative H&E staining of distal femur sections from Sham, Model, and Rop groups (scale bar: 100 μm).

**Figure S2.** (A) Tartrate-resistant acid phosphatase (Trap) staining of distal femur sections and (B) quantitative analysis of osteoclast area fraction (scale bar: 100 μm).

**Figure S3.** Representative micro-CT reconstructions of trabecular bone microstructure in the distal femur, shown in both isometric and top-down views.

**Figure S4.** Quantitative analysis of Nissl body number in the spinal cord.

**Figure S5.** NGF immunofluorescence in spinal cord tissue. (A) Representative single-channel immunofluorescence images of spinal cord tissue from Sham, Model, and Rop groups. Spinal cord sections are labeled with NGF (red, neuronal activation marker) and DAPI (blue, nuclear counterstain) (scale bar: 100 μm). (B) Quantification of mean fluorescence intensity of NGF in spinal cord tissue.

**Figure S6.** CGRP and c-Fos immunofluorescence in spinal cord tissue. (A) Representative single-channel immunofluorescence images of spinal cord tissue from Sham, Model, and Rop groups. Spinal cord sections are labeled with CGRP (green, neuronal marker), c-Fos (red, neuronal activation marker), and DAPI (blue, nuclear counterstain) (scale bar: 50 μm). (B-C) Quantification of mean fluorescence intensity of (B) CGRP and (C) c-Fos in spinal cord tissue.

**Figure S7.** Representative single-channel immunofluorescence images of primary neurons treated with PBS, Rop, TA and TA+Rop. Neurons are labeled with β-tubulin Ⅲ (green, neuronal marker), c-Fos (red, neuronal activation marker), and DAPI (blue, nuclear counterstain) (scale bar: 50 μm).

**Figure S8.** Representative fluorescence microscopy images of intracellular reactive oxygen species (ROS) staining in DRG neurons (scale bar: 100 μm).

**Figure S9.** Photographs of lyophilized PTA polymer and PTA@Rop nanogels.

**Figure S10**. GPC characterization of PTA. The synthesized PTA exhibited a number-average molecular weight (Mn) of 1,406 Da and a dispersity (Đ) of 1.364.

**Figure S11.** Local retention and PTA@Rop after in situ administration. (A) Representative longitudinal IVIS images of the tumor-bearing femur after administration of FITC-labeled PTA@Rop. (B) Quantification of fluorescence intensity at the leg and femur administration site. (C) HPLC quantification of Rop retained at the tumor-bearing femur after administration of free Rop or PTA@Rop. Data are presented as mean ± SD.

**Figure S12.** Comparison of reduction-responsive PTA@Rop with non-redox-responsive PLGA@Rop. (A) Hydrodynamic size and PDI. (B) Rop-release profiles in PBS with or without 10 mM GSH at 37 °C. Data are presented as mean ± SD.

**Figure S13.** Representative single-channel immunofluorescence images of primary neurons treated with PBS, Rop, TA+Rop, and PTA@Rop. Neurons are labeled with β-tubulin III (green, neuronal marker), c-Fos (red, neuronal activation marker), and DAPI (blue, nuclear counterstain) (scale bar: 50 μm).

**Figure S14.** Representative fluorescence microscopy images of intracellular reactive oxygen species (ROS) staining in DRG neurons (scale bar: 100 μm).

**Figure S15.** Representative H&E staining of plantar wound skin tissue from the incisional pain model (scale bar: 100 μm).

**Figure S16.** Body weight changes of mice in different groups over the 13-day experimental period. Values are presented as the percentage of initial body weight.

**Figure S17.** Analgesic efficacy of PTA@Rop in an intrafemoral 4T1 breast-cancer cell-derived BCP model. (A) Experimental timeline showing intrafemoral inoculation, drug administration, behavioral assessment, and sample collection. (B) Mechanical withdrawal thresholds and (C) thermal withdrawal latencies at the indicated time points. Data are presented as mean ± SD (n = 6). Statistical significance was determined using two-way repeated-measures ANOVA followed by Tukey’s multiple-comparisons test. ^*^p < 0.05, ^**^p < 0.01, and ^***^p < 0.001.

**Figure S18.** Gait pressure analysis of the right forelimb (RF), right hindlimb (RH), left forelimb (LF), and left hindlimb (LH) in mice from different groups. The colored bars represent the stance phase duration of each limb over time.

**Figure S19.** Quantitative analysis of key gait parameters of the left hindpaw (LH) across different groups. (A) Mean paw print intensity, (B) swing phase duration, (C) stance phase duration, and (D) stride length. ^*^*P* < 0.05, ^**^*P* < 0.01, ^***^*P* < 0.001, ns, not significant.

**Figure S20.** Representative histological and immunohistochemical analysis of mouse femurs. (A) H&E staining and tartrate-resistant acid phosphatase (TRAP) staining of femur sections from Sham, Model, Rop, TA, TA+Rop, and PTA@Rop groups (scale bar: 100 μm). (B) Quantitative analysis of the TRAP-positive area fraction.

**Figure S21.** Representative micro-CT reconstructions of trabecular bone microstructure in the distal femur, shown in both isometric and top-down views.

**Figure S22.** Quantitative analysis of Nissl body number in the spinal cord.

**Figure S23.** NGF immunofluorescence in spinal cord tissue. (A) Representative single-channel immunofluorescence images of spinal cord tissue from Sham, Model, Rop, TA, TA+Rop and PTA@Rop groups. Spinal cord sections are labeled with NGF (red, neuronal activation marker) and DAPI (blue, nuclear counterstain) (scale bar: 100 μm). (B) Quantification of mean fluorescence intensity of NGF in spinal cord tissue.

**Figure S24.** CGRP and c-Fos immunofluorescence in spinal cord tissue. (A) Representative single-channel immunofluorescence images of spinal cord tissue from Sham, Model, Rop, TA, TA+Rop and PTA@Rop groups. Spinal cord sections are labeled with CGRP (green, neuronal marker), c-Fos (red, neuronal activation marker), and DAPI (blue, nuclear counterstain) (scale bar: 50 μm). (B-C) Quantification of mean fluorescence intensity of (B) CGRP and (C) c-Fos in spinal cord tissue.

**Figure S25.** PTA@Rop reduces ROP-induced inflammatory gene expression. The relative mRNA expression levels of *Tnf*, *Il1b*, *Il6*, *Ccl2*, and *Nfkbia* in the collected tissues were detected by RT-qPCR. The dotted line represents the expression level of the Sham group. Data are presented as mean ± SD (n = 3). Statistical analysis was performed using one-way ANOVA followed by Tukey’s multiple-comparison test. ^**^*p* < 0.01 and ^***^*p* < 0.001.

**Figure** **S26.** Repeated-dose *in vivo* safety evaluation of PTA@Rop. (A) Body-weight changes during the 14-day treatment period. (B) Organ coefficients of the heart, liver, spleen, lung, and kidney after treatment. Data are presented as mean ± SD (n = 3).

### Table S1**. Radiographic scoring criteria for cancer-induced bone destruction**

| **Score** | **Radiographic manifestation** |
| --- | --- |
| 0 | Normal; no signs of bone destruction |
| 1 | Mild; 1–3 small bone destruction pits |
| 2 | Moderate; 4–6 bone pits and loss of medullary bone |
| 3 | Severe; medullary bone loss and cortical bone erosion |
| 4 | Severe + unilateral defect; full-thickness unicortical bone loss |

Note: Each femur was divided into distal and proximal segments for independent scoring, and the average value was used for statistical analysis.

**Table S2. Characterization of PTA@Rop.**

| Batch | particle size  (nm) | polydispersity index (PDI) | drug loading content (DL%) | encapsulation efficiency (EE%) |
| --- | --- | --- | --- | --- |
| 1 | 568.3 | 0.7191 | 7.9 | 69.125 |
| 2 | 579.4 | 0.7066 | 9.5 | 83.1 |
| 3 | 660.9 | 0.7652 | 7.3 | 63.875 |
| Mean ± SD | 602.9 ± 50.6 | 0.7303 ± 0.0309 | 8.2 ± 1.1 | 72.0 ± 9.9 |

**Table S3.** **RT-qPCR primer sequences**

| **Gene** | **Direction** | **Sequence (5'→3')** | **Amplicon (bp)** |
| --- | --- | --- | --- |
| ***GAPDH*** | Forward | TGATGGGTGTGAACCACGAG | 152 |
| ***GAPDH*** | Reverse | AGTGATGGCATGGACTGTGG | 152 |
| ***Tnf*** | Forward | CACAGAAAGCATGATCCGCG | 211 |
| ***Tnf*** | Reverse | ACTGATGAGAGGGAGGCCAT | 211 |
| ***Il1b*** | Forward | AACGAGGCTTATGTGCACGA | 241 |
| ***Il1b*** | Reverse | TCAACACGCAGGACAGGTAC | 241 |
| ***Il6*** | Forward | CAGCCATCCTCCCCCATTTT | 278 |
| ***Il6*** | Reverse | AGACCCTTGCACAACACCAA | 278 |
| ***Ccl2*** | Forward | ACCTTCATTCCCCAAGGGCT | 200 |
| ***Ccl2*** | Reverse | CACTTCTGCTTGGGGTCAGC | 200 |
| ***Nfkbia*** | Forward | TGCACTTGGCCATCATCCAT | 389 |
| ***Nfkbia*** | Reverse | AGCACCCAAGGACACCAAAA | 389 |
